# Riboflavin-Vancomycin Conjugate Enables Simultaneous Antibiotic Photo-Release and Photodynamic Killing against Resistant Gram-Positive Pathogens

**DOI:** 10.1021/jacsau.3c00369

**Published:** 2023-10-24

**Authors:** Bethany Mills, Alex Kiang, Syam Mohan P. C. Mohanan, Mark Bradley, Maxime Klausen

**Affiliations:** † EaStCHEM School of Chemistry, University of Edinburgh, David Brewster Road, EH9 3FJ Edinburgh, U.K.; ‡ Translational Healthcare Technologies group, Centre for Inflammation Research, Queen’s Medical Research Institute, University of Edinburgh, 47 Little France Crescent, Edinburgh EH16 4TJ, U.K.

**Keywords:** photodynamic therapy, photolabile protecting groups, uncaging, antimicrobial resistance, antibiotic, ESKAPE pathogens

## Abstract

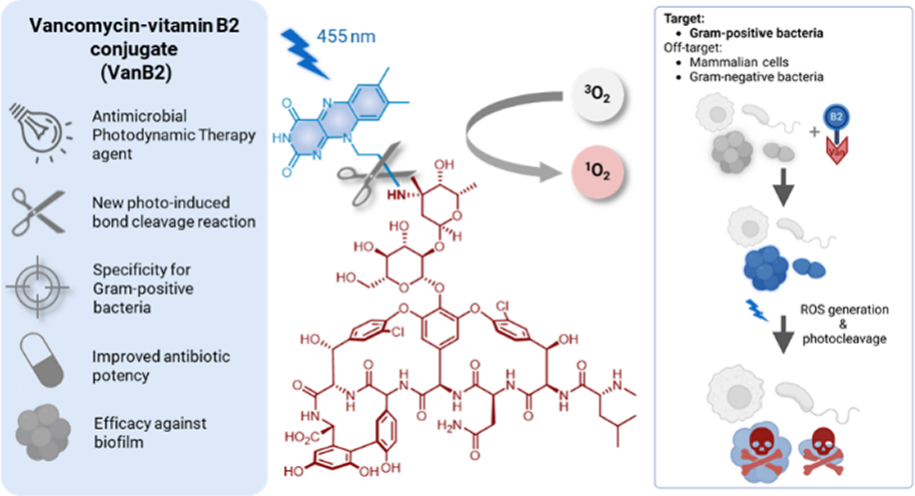

Decades of antibiotic misuse have led to alarming levels of antimicrobial resistance, and the development of alternative diagnostic and therapeutic strategies to delineate and treat infections is a global priority. In particular, the nosocomial, multidrug-resistant “ESKAPE” pathogens such as Gram-positive methicillin-resistant *Staphylococcus aureus* (MRSA) and vancomycin-resistant *Enterococcus spp* (VRE) urgently require alternative treatments. Here, we developed light-activated molecules based on the conjugation of the FDA-approved photosensitizer riboflavin to the Gram-positive specific ligand vancomycin to enable targeted antimicrobial photodynamic therapy. The riboflavin-vancomycin conjugate proved to be a potent and versatile antibacterial agent, enabling the rapid, light-mediated, killing of MRSA and VRE with no significant off-target effects. The attachment of riboflavin on vancomycin also led to an increase in antibiotic activity against *S. aureus* and VRE. Simultaneously, we evidenced for the first time that the flavin subunit undergoes an efficient photoinduced bond cleavage reaction to release vancomycin, thereby acting as a photoremovable protecting group with potential applications in drug delivery.

## Introduction

The emergence of antimicrobial-resistant (AMR) and multidrug-resistant (MDR) bacteria has been exacerbated by the misuse and overuse of antibiotics and pose a major threat to human health.^1,2^ In particular, the emergence of the nosocomial, MDR “ESKAPE” pathogens (*Enterococcus spp.*; *Staphylococcus aureus*; *Klebsiella pneumoniae*; *Acinetobacter baumanii*; *Pseudomonas aeruginosa* and *Enterobacter spp.*),^3^ which have been designated as “critical” and “high priority” for the development of alternative treatments by the World Health Organization (WHO),^4^ is a significant cause for concern. Importantly, a dozen novel antibiotics have been approved in the past five years,^5^ however, resistance development remains a fast-paced issue global issue,^6,7^ and there is a constant and urgent need to develop approaches that move beyond the classical bactericidal pathways.^8^ Photodynamic therapy (PDT) offers great potential in this regard.^8−10^ PDT relies on the administration of a photosensitizer (PS), which generates a range of reactive oxygen species (ROS) upon absorption of specific wavelengths of light.^11−13^ Following absorption, the PS molecule undergoes intersystem-crossing from a singlet excited state to a long-lived triplet state, which enables the generation of ROS in the form of hydroxyl radicals (Type I photoprocess) and/or singlet oxygen (Type II photoprocess). The high and localized toxicity of these short-lived species has been widely exploited for the treatment of cancer,^14^ skin,^15,16^ and oral diseases.^17,18^ However, applications for PDT have the potential to extend far beyond these and are particularly attractive in the context of infection, especially when aimed toward topical infections amenable to light delivery such as those of the skin (wounds, burns, and diabetic foot ulcers), cornea, surgical sites, or the oral cavity, for which Gram-positive bacteria including methicillin-resistant *S. aureus* (MRSA) and vancomycin-resistant *Enterococcus spp.* (VRE) remain clinical challenges.

Antimicrobial PDT (aPDT) has the potential to become a sustainable alternative to standard antibiotic treatment as pathogens are considered unlikely to develop resistance mechanisms to the lethal and fast-acting ROS generated by the PS.^19^ The challenge however lies in the design of photosensitizing drugs with a high therapeutic index to limit off-target phototoxicity against healthy mammalian cells.^10,20^ Cationic PS which bind nonspecifically to negatively charged microbial membranes have been successfully deployed in aPDT.^21−23^ However, clinical aPDT remains at an early stage of development compared to anticancer PDT,^24,25^ partly because of poor pathogen selectivity.

Among the strategies to augment photosensitizing drug specificity is the covalent conjugation of PS molecules to pathogen-specific ligands to enable direct binding to microbes. We have previously demonstrated targeted aPDT eradication of Gram-negative bacteria with a probe based on a methylene blue PS conjugated to a modified polymyxin B scaffold^26^ allowing the generation of ROS in direct proximity of the pathogens. Here, we aimed to expand the aPDT toolbox by designing and synthesizing a novel, complementary aPDT probe to target Gram-positive bacteria and validate it against pathogens including MRSA and VRE. Our Gram-positive-specific aPDT agent was designed as a covalent conjugate of the glycopeptide antibiotic vancomycin as the binding ligand and riboflavin (vitamin B2) as the PS. Riboflavin was selected due to its high biocompatibility, low cost, and FDA-approved status while its singlet oxygen quantum yield (Φ_Δ_) of 0.54^27^ shows high ROS generation efficiency. The riboflavin-vancomycin conjugate led to the complete photodynamic-mediated killing of MDR Gram-positive infections after only 20 min of illumination. During our investigation, we also discovered for the first time that the flavin chromophore acts as a photoremovable protecting group (PPG)^28,29^ for amine groups via photocleavage of its side chain, which here led to concomitant vancomycin release during the course of the aPDT treatment. Although the photosensitivity of vitamin B2 via radical side-chain oxidation has been known for decades,^30,31^ this is the first example of a modified riboflavin showing properties as a PPG for amines, which makes it a useful scaffold for applications in both PDT and light-mediated drug-delivery.

## Results and Discussion

### Chemical Synthesis and Characterization

**Scheme 1:**
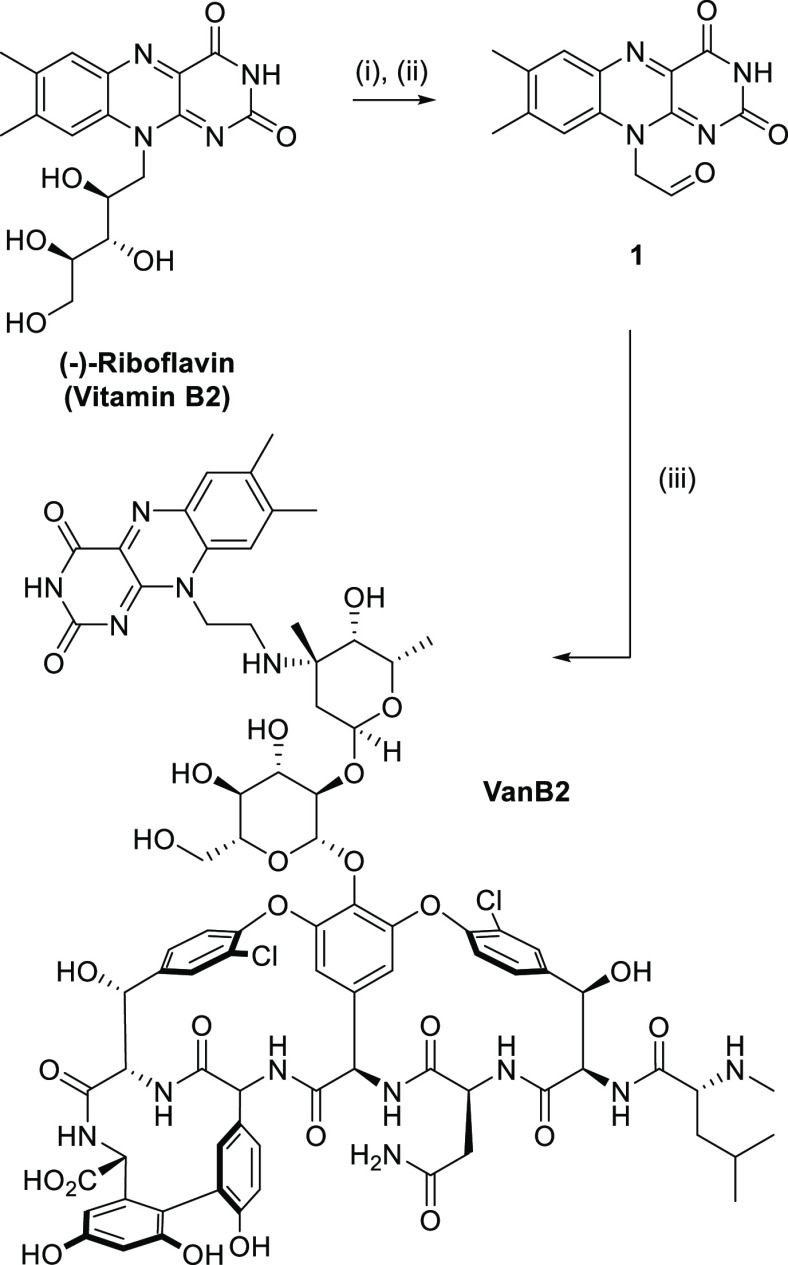
Synthesis of VanB2 Reagents and conditions: (i) NaIO_4_, H_2_O, r.t., 16 h. (ii) Toluene, reflux, 4 h. (iii) Vancomycin hydrochloride, DIPEA, DMF, NaBH_3_CN, MeOH, TFA, r.t., 16 h.

In order to achieve high Gram-positive bacteria selectivity, the targeted photosensitizing agent was designed by modifying vancomycin on its amino-glycan moiety by reductive amination, a strategic modification used to tune the potency of glycopeptides against resistant Gram-positive bacteria, as demonstrated in derivatives such as oritavancin.^32^ The preparation of the PDT probe was carried out in an efficient two-step synthesis starting from vitamin B2 (Scheme 1). The polyol sidechain of riboflavin was cleaved under oxidative conditions to yield aldehyde **1**, which was directly attached to vancomycin hydrochloride by reductive amination.^33^ This sequence afforded the target aPDT probe **VanB2** on a gram scale and the new light-activated probe was fully characterized by NMR, HPLC, HRMS, and MALDI-TOF MS (see the SI).

**Figure 1:**
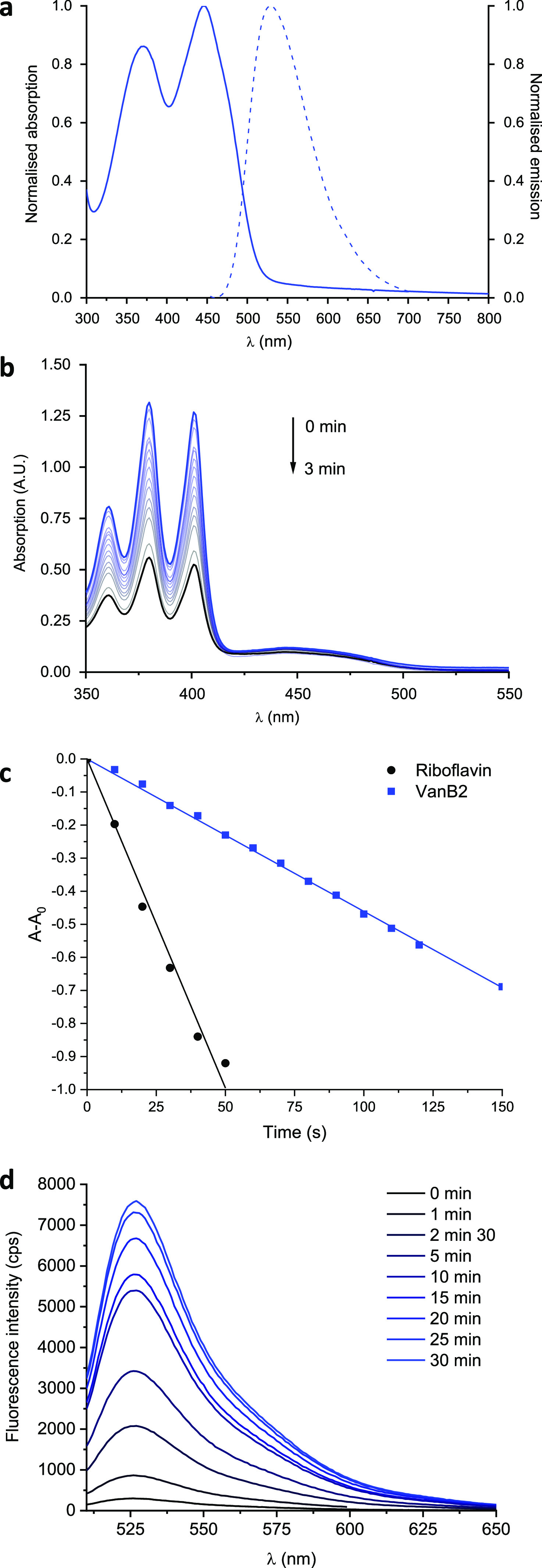
Optical properties and singlet oxygen generation of the new PS agents in PBS. (a) Normalized absorption (continuous) and emission (dashed) spectra of VanB2. Fluorescence spectra were recorded at the maximum of excitation of the riboflavin band according to the values reported in Table 1. (b) Evolution of the absorption spectrum of a PBS solution containing the ^1^O_2_ sensor ABMDMA and VanB2 upon excitation at 470 nm over 3 min. (c) Kinetics of the decrease in absorbance of the ABMDMA sensor at 380 nm over time during irradiation in the presence of riboflavin (reference, black circles) and VanB2 (blue squares). (d) Evolution of the fluorescence spectrum of a PBS solution containing the ROS sensor DHR123 and VanB2 upon excitation at 470 nm over 30 min.

The absorption and emission properties of the aPDT probe **VanB2** (Table 1) were in accordance with unconjugated riboflavin, with a large absorption band at 444 nm and an absorption coefficient of 1.2 × 10^4^ M^–1^ cm^–1^ at this wavelength in PBS and a second weaker absorption band at 370 nm. Similar properties were observed in MeOH and DMSO, with only a moderate solvatochromic behavior (Figure S1). The **VanB2** conjugate retained a residual green emissive character (Φ_f_ = 0.02), albeit much less bright than unmodified riboflavin (Φ_f_ = 0.27).^34^ This fluorescence was slightly blue-shifted but brighter in MeOH and DMSO. The generation of ROS was investigated via irradiation of the probe (10 μM) at 470 nm (4.0 mW/cm^2^) in the presence of ROS chemical traps and comparing the results with unmodified riboflavin under the same experimental conditions. Irradiation in the presence of the water-soluble ^1^O_2_ sensor 9,10-anthracenediyl-bis(methylene)dimalonic acid (ABMDMA, 100 μM) led to a decrease of its absorbance at 380 nm (Figures 1, S2 and S3), indicating the formation of the corresponding deep-UV absorbing endoperoxide generated after reaction with singlet oxygen (Scheme S1). The ABMDMA sensor was selected both for its solubility in water and for its absorption located below 400 nm, which prevents absorption of the 470 nm light by the sensor during the assay.

**Table 1:** Photophysical Properties of VanB2 in Different Solvents

solvent	λ_abs_^max^ (nm)	ε^max^ (M^–1^ cm^–1^)	λ_em_^max^ (nm)	Stokes shift (cm^–1^)	Φ_f_^a^	Φ_Δ_^b^
MeOH	439	1.0 × 10^4^	512	3247	0.23	0.71
DMSO	442	1.3 × 10^4^	510	3017	0.08	–c
PBS	444	1.2 × 10^4^	528	3583	0.02	0.16

a Fluorescence quantum yield measured upon excitation at the maximum of the riboflavin absorption band, relative to fluorescein in NaOH 0.1 M (Φ_f_ = 0.90).

b Singlet oxygen quantum yield, determined by a relative measurement method; upon irradiation at 470 nm (4.0 mW/cm^2^) in the presence of the singlet oxygen sensor ABMDMA (100 μM) and comparison with (−)-Riboflavin under identical conditions (see the SI for details).

c Only degradation of the riboflavin ring was observed.

The kinetics and quantification of the ^1^O_2_ trapping by the sensor were monitored (Figure 1, c), which allowed the determination of the relative rates of ^1^O_2_ generation and the corresponding ^1^O_2_ quantum yields Φ_Δ_ (see the SI). The absorbance band of ABMDMA disappeared after only 40 s in the presence of the reference riboflavin, but only decreased by 12% in the presence of **VanB2**, leading to a comparative Φ_Δ_ of 0.16. Type-I photoprocesses were also investigated using dihydrorhodamine 123 (DHR123, 10 μM in water) and monitoring the increase in fluorescence at 526 nm upon oxidation of DHR123 to rhodamine 123 (Scheme S2), which led to a similar pattern (Figures 1d, S4 and S5). This is the first indication that the modification of the riboflavin sidechain strongly affects the relaxation processes from the excited state. Since the π-conjugated system of the PS was not modified during the synthesis, intramolecular interactions such as excited-state hydrogen bonding are likely to be the cause of the drop of Φ_Δ_. Additionally, in all solvents, the absorption band of the flavin unit decreased during the course of the irradiation (Figures 1, S2 and S3), indicating that the conjugated structure of the flavin chromophore is likely affected by a photodegradation reaction (see below). Meanwhile, these assays also showed that ^1^O_2_ generation promoted by the probe was highly sensitive to the environment, with 4-fold higher Φ_Δ_ values in MeOH compared to PBS (Table 1 and Figure S3). Interestingly, in DMSO, which is known to react with singlet oxygen to form dimethylsulfone,^35^ the irradiation experiments evidenced clearly the photodegradation of the riboflavin unit, even without oxidation of the ABMDMA sensor (Figure S3). Noteworthy, the degradation of the flavin ring was also observed for the reference compound riboflavin in DMSO and MeOH solutions. It was not clearly evidenced in PBS in the time frame of the irradiation, although it can be assumed that it would be observed with prolonged irradiation times. The degradation of **VanB2** under blue light was then further investigated.

### Vancomycin Release via Riboflavin Photocleavage

**Figure 2:**
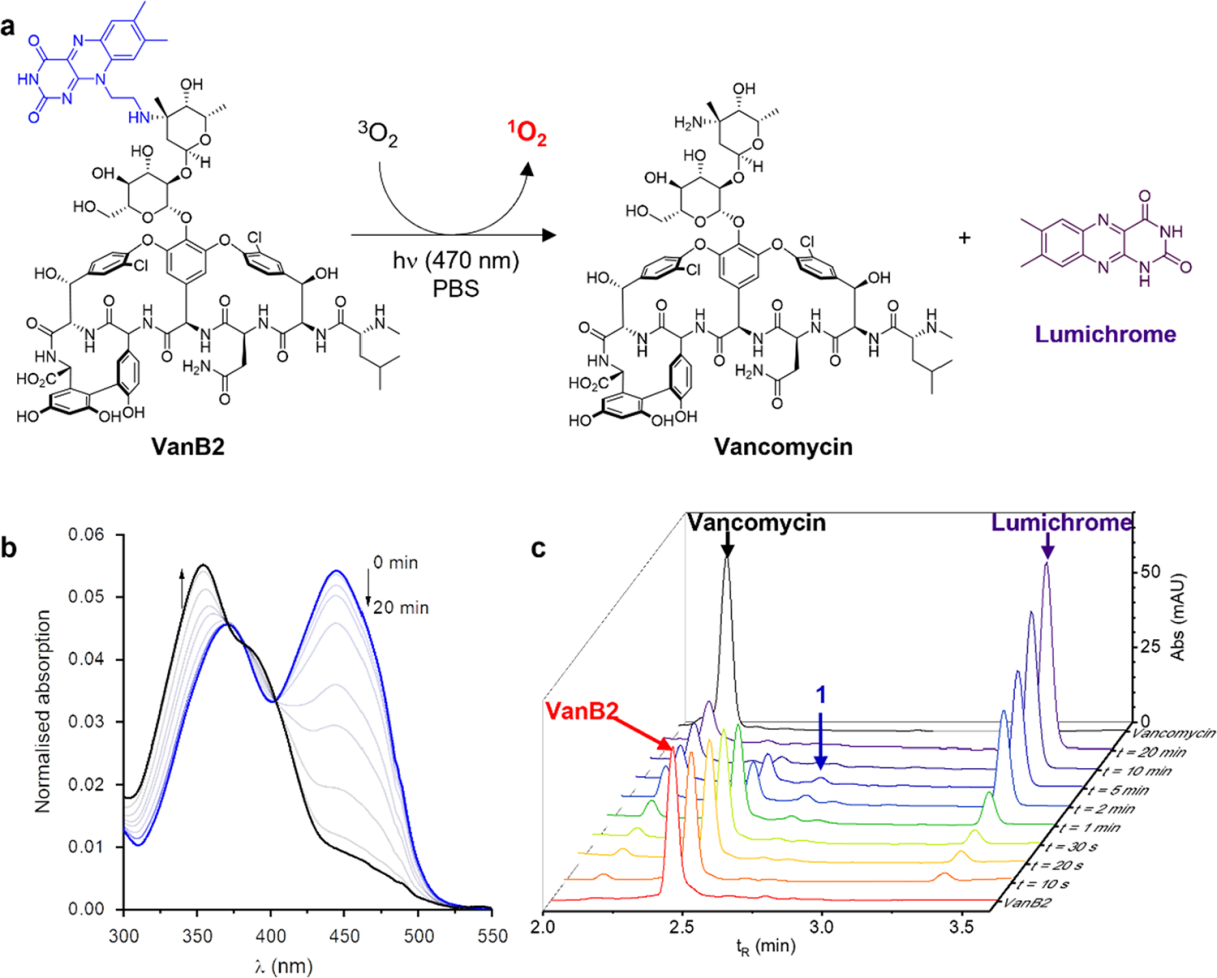
(a) Photolysis reaction occurring during the irradiation of VanB2, with the independent generation of singlet oxygen and the cleavage of the riboflavin “protecting group”. (b) Evolution of the absorbance spectrum during 20 min of irradiation (470 nm) of VanB2 in PBS, showing the disappearance of the riboflavin band at 440 nm and the appearance of an absorbance at 350 nm due to lumichrome. (c) HPLC time course (detection at 254 nm) of the photolysis products of VanB2 (2.5 μM, 470 nm) showing the formation of vancomycin and lumichrome with intermediate formation of aldehyde 1.

The irradiation experiments performed with **VanB2** evidenced a “self-destructive” character under blue light, with the absorbance of the flavin band of **VanB2** in PBS (444 nm) decreasing by 50% after 3 min of irradiation while UV absorption (350 nm) increased, with an isosbestic point at 403 nm (Figure 2b). This indicated that the π-conjugated structure of the riboflavin unit was likely modified during the course of the irradiation, and that the stoichiometry of this reaction remains unchanged. In order to understand the structural modifications at play in this apparent bleaching mechanism, the photolysis of **VanB2** was performed in PBS, monitoring the reaction by UV–vis spectroscopy and HPLC-MS. During the course of the irradiation, HPLC showed the disappearance of the peak corresponding to **VanB2**, and the appearance of two photoproducts at 2.16 and 3.37 min (Figure 2c) that were attributed, respectively, to vancomycin and lumichrome, a known byproduct of riboflavin photolysis, via the intermediate aldehyde **1**.^30,31^ Products were identified both by HPLC–MS (Figure S6) and individual injection of the corresponding standards. This confirms that the linker between vancomycin and riboflavin is cleaved in a photochemical cascade, thereby releasing the vancomycin scaffold (Figure 2a). The concomitant formation of lumichrome, a blue-shifted flavin derivative (λ_abs_^max^ = 385 nm), as a byproduct of the cleavage is also in accordance with the UV–vis monitoring of the photolysis (Figure 2b) showing a decrease in the absorbance of **VanB2** at 440 nm and an increase in the intensity of the 380 nm band. Control experiments confirmed that solutions of lumichrome do not generate singlet oxygen with blue light (Figure S7),

Monitoring the release of the caged vancomycin upon irradiation by HPLC allowed the determination of several key parameters of the photolytic reaction. HPLC quantification (Figure S8) showed that the chemical yield of the photorelease of vancomycin was >90%, with the photocleavage reaction following first-order kinetics in PBS (Figure S9). The rate of the reaction was essentially unaffected when the photolysis was performed under anaerobic conditions (i.e., under continuous nitrogen flow, see the SI), proving the reaction to be oxygen-independent, and therefore not singlet-oxygen mediated.

Kinetic analysis allowed determination of the photochemical quantum yield of this new uncaging reaction. The uncaging quantum yield (Φ_u_) is defined as the number of “caged” molecules released per 100 photons absorbed, which is indicative of the photochemical efficiency of the bond cleavage.

The Φ_u_ value is known to be directly related to the kinetics of the uncaging reaction (see the SI) and to the total irradiation intensity at 470 nm, which was measured using the potassium ferrioxalate chemical actinometry method (see the SI).^36^ Using the rate constant determined from kinetic analysis, the photochemical quantum yield Φ_u_ of vancomycin uncaging could be determined. A value of Φ_u_ = 0.0027 was obtained, which was in good agreement with the photochemical-dissociation quantum yield reported for the cleavage of the side chain of natural riboflavin in phosphate buffer (Φ_diss_ = 0.0078^37^).

The photocleavable character evidenced in **VanB2** is likely the result of a triplet diradical formed in the excited state. In natural vitamin B2, this diradical evolves toward the oxidative cleavage of the poly-ol side-chain and the formation of aldehyde **1**.^30,31^ The formylmethyl moiety can then be further cleaved at neutral and acidic pH to yield lumichrome and glycolaldehyde.^31^ Although further mechanistic investigation is required, the transient formation of aldehyde **1** evidenced by HPLC-MS indicates that **VanB2** likely follows a similar diradical pathway, where the ethylamine linker releases the caged amine instead of the hydroxyl side chain of unmodified riboflavin. In the present case, the diradical cascade may initially involve the formation of an imine, which would then be hydrolyzed to aldehyde **1**, and vancomycin. Lumichrome and glycolaldehyde would then be formed according to reported degradation mechanisms.^31^ Additional photolysis experiments were performed in deuterated solvents, following the evolution of the ^1^H NMR spectra of **VanB2** over the course of the irradiation. Interestingly, the photocleavage did not proceed in pure D_2_O, only starting upon the addition of a trace of acid. This indicates that the release mechanism in aqueous media is likely pH-dependent, which supports the hypothesis of it proceeding via possible imine hydrolysis. Photolysis in DMSO-*d*_6_ (Figure S10) confirmed the formation of free vancomycin (representative singlet at 7.86 ppm, in addition to the rest of the signals matching the reported assignment in DMSO-*d*_6_^38^) and the simultaneous release of lumichrome (singlets appearing at 7.90 and 7.71 ppm) during the course of the irradiation. No further reaction intermediates or byproducts were identified.

These findings show that the flavinylethyl scaffold behaves as a new light cleavable protecting group and is able to uncage a bioactive molecule via a protected amine group under blue light irradiation. This indicates that a stoichiometric dose of vancomycin will be released in parallel to the PDT treatment, which opens avenues into combination therapy strategies where the release of multiple payloads can be envisioned. In addition, solutions of **VanB2** in PBS also proved to be very robust in the dark, which is a crucial point in drug photo-activation strategies. No modification of the absorption spectra or HPLC traces was observed after 1 year of storage at −20 °C, and solutions of the compound stored at room temperature were still 82% pure after a month (Figure S11). This indicates that no competitive dark cleavage or degradation occurs in water and is crucial for potential translation to clinical applications.

### VanB2 Killing of Gram-Positive Bacteria

**Figure 3:**
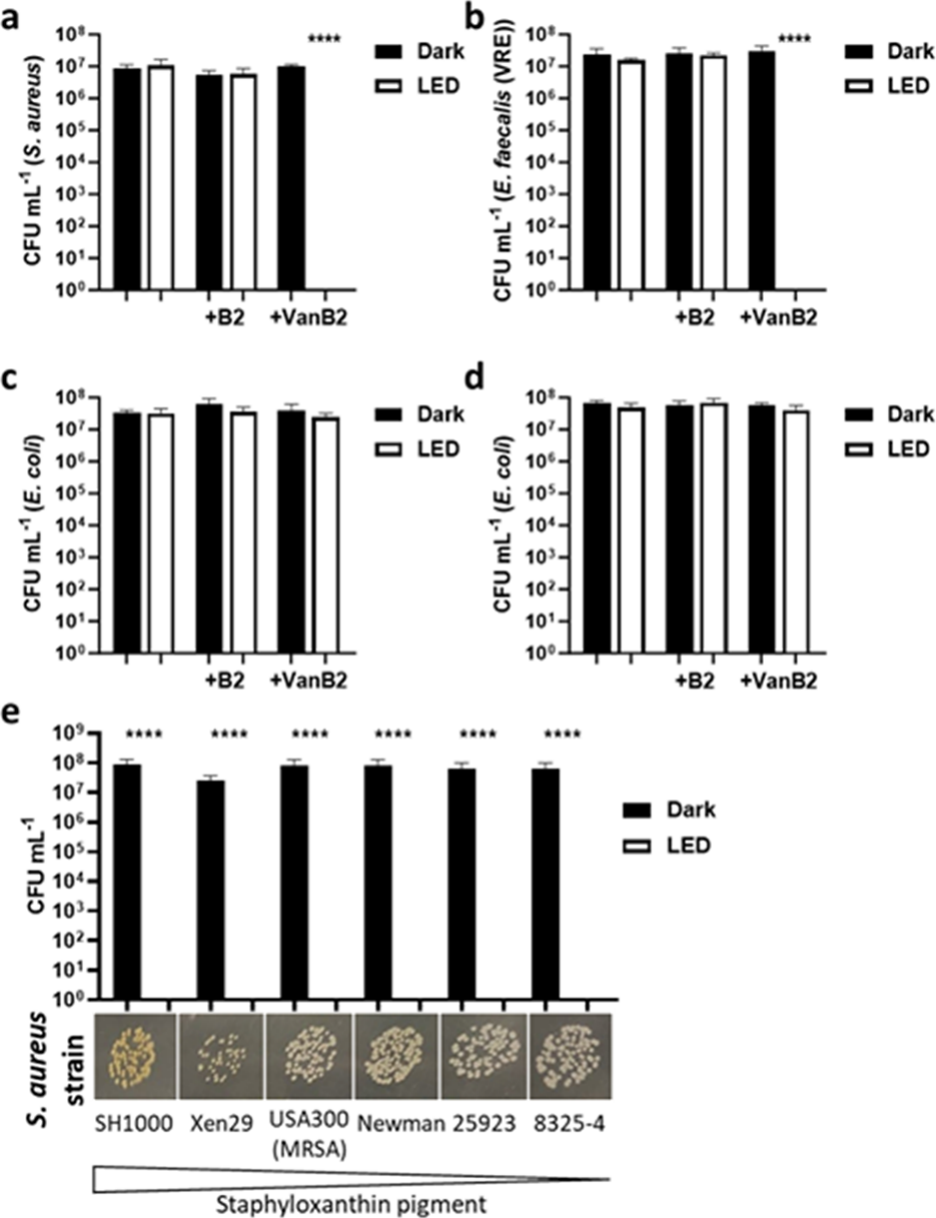
Colony forming units (CFU) of bacteria following aPDT treatment with VanB2 or riboflavin (B2). Gram-positive bacteria (a) *S. aureus*; (b) Vancomycin resistant *E. faecalis*; and (c) Gram-negative bacteria *E. coli* were incubated with VanB2 or B2 (5 μM) for 10 min prior to removal of excess compound followed by 20 min illumination (455 nm, 30 mW/cm^2^). Controls were maintained in the dark with or without compound. All samples were plated for colony forming units (CFU) to determine bacterial survival. (d) *E. coli* was treated as in (c) without removal of excess compound prior to illumination. (e) CFU and photograph of *S. aureus* strains with differential expression of staphyloxanthin (orange-yellow pigment) following aPDT with VanB2 (and dark controls). Error bars show s.e.m., (a–d) analyzed one-way ANOVA with comparison to (a–d) bacteria-only control or (e) dark control: ****P < 0.0001. *n* = 3.

The sensitivity, specificity and phototoxic antibacterial properties of **VanB2** against target (Gram-positive bacterial strains *S. aureus* and vancomycin-resistant *E. faecalis*), and off-target (Gram-negative *E. coli*) bacteria were determined and compared to equimolar concentrations of natural riboflavin and vancomycin. The compounds (5 μM) were incubated with the bacteria for 10 min (washing away excess compound), followed by either 20 min illumination (455 nm LED, 30 mW/cm^2^, Figure S12) or maintained in the dark to prevent photoactivation of the probe. Following PDT treatment with **VanB2**, no Gram-positive bacterial colonies were recovered when plated for colony forming units (CFUs), marking a 7-log reduction (*P* < 0.0001) in CFU mL^–1^ compared to **VanB2** labeled bacteria maintained in the dark, riboflavin controls (with and without illumination), and PS-free controls (Figure 3). *E. coli* remained unaffected by any treatment, even with **VanB2** in excess (Figure 3d). This suggests that any ROS generated within the surrounding microenvironment was too diffuse to yield an off-target biological response, demonstrating the importance of locking the PS onto the target pathogen.

A dose response of **VanB2** phototoxicity against *S. aureus* determined that 0.63 μM caused complete killing following illumination, while the effect was lost at 0.16 μM, with an intermediate level of killing observed at 0.31 μM (Figure S13). There was no killing induced by riboflavin, or the vancomycin controls, in the dark or following illumination, even at 10 μM, on the short exposure involved for the aPDT experiments (i.e., 30 min). On this short time scale, *S. aureus* remained unaffected by the contact with vancomycin despite being sensitive to the antibiotic at concentrations less than 3 μM (<5 μg mL^–1^, determined by conventional minimum inhibitory concentration (MIC) assay (Table S1)).

When applied in nonphotodynamic situations (i.e., conventional antibiotic action over 15 h), the antibiotic activity of **VanB2** (Figure S14) was also enhanced over vancomycin. While 10 μM vancomycin caused complete growth inhibition of *S. aureus*, 1 μM **VanB2** was enough to totally inhibit the growth rate of *S. aureus* through its antibiotic activity (in the dark). This enhanced activity can be explained by the introduction of the aromatic riboflavin subunit on the vancosamine position which is known to enhance potency and is consistent with the reduced MIC observed in other arylated vancomycin derivatives (e.g., oritavancin).

We further sought to characterize the aPDT and antibiotic potency of **VanB2** against bacteria strains expressing vancomycin resistance. The vancomycin-resistant *E. faecalis* strain utilized here possessed so-called “Van-B mediated” resistance, which confers a modified peptidoglycan structure (where d-Ala-d-Ala is replaced by d-Ala-d-Lac in peptidoglycan cell-wall precursors)^39^ reducing the binding affinity of vancomycin.^40^ The vancomycin resistance was confirmed with an MIC value between 34 and 68 μM (Table S1).

Despite this, **VanB2** proved to be a proficient aPDT agent against the VRE strain. Complete *E. faecalis* killing was achieved using 1.25 μM of the probe, within 20 min of illumination, rendering a 7-log reduction in cell viability (*P* < 0.0001) (Figures 3b and S13). This was twice the concentration required for the same CFU reduction of *S. aureus* under the same experimental conditions, which suggests a modest reduction in **VanB2** susceptibility, possibly due to a reduced binding affinity consistent with the mechanism of vancomycin resistance. Additionally, the nonlight-based, antibiotic potency of **VanB2** against vancomycin-resistant strains proved to be significant, completely preventing growth of VRE over 15 h at 10 μM, and partially slowing it at 1 μM (Figure S14). This indicates that the vancosamine modification with riboflavin is a powerful strategy not only to strongly increase the potency of vancomycin derivatives but also to bypass Van-B resistance mechanisms.

Time-to-kill was also examined for both *S. aureus* and *E. faecalis* labeled with 5 μM **VanB2**. Complete killing of *S. aureus* was achieved within 2 min of illumination with the LED setup (455 nm, 30 mW cm^–2^) conferring an irradiation dose of 3.6 J cm^–2^, whereas complete killing of *E. faecalis* required 9 J cm^–2^ (5 min illumination) (Figure S13c), indicating that the PS was extremely efficient, even with low irradiation doses.

While characterizing **VanB2** aPDT efficacy in planktonic-state bacteria is an essential measure of activity, when considering potential applications for clinical translation, assessment in more complex models is imperative. We first assessed the aPDT potency of **VanB2** against a bacterial lawn grown on agar (nutrient-rich) plates. **VanB2** was added to the center of each lawn and illuminated at 455 nm (or maintained in the dark as controls). For both *S. aureus* and *E. faecalis,* growth exclusion zones from the area concurrently receiving **VanB2** and illumination were apparent (Figure S15). *S. aureus* exhibited only a modest growth reduction with 5 μM of compound, but complete killing at 10 μM with LED in this model. Moderate dark toxicity was observed at 10 μM **VanB2** and complete killing at 25 μM, demonstrative of the antimicrobial effect of **VanB2**. Interestingly, in this model, complete killing of *E. faecalis* was observed with as little as 5 μM of **VanB2**, and no dark toxicity was observed at any concentration. Areas of the bacterial lawn within the illumination zone but without **VanB2** were unaffected.

The aPDT effect of **VanB2** against bacterial biofilms with *S. aureus* and *E. faecalis* was then examined. Biofilms remain a major therapeutic challenge due to their inherent resistance to antibiotics due to poor drug penetration and lower metabolic activity, often conferring a 10–1000-fold increase in antimicrobial MIC compared to planktonic bacteria.^41^ Consequently, aPDT against bacterial biofilm often requires higher concentrations of PS and a longer duration of illumination.^42^

**Figure 4:**
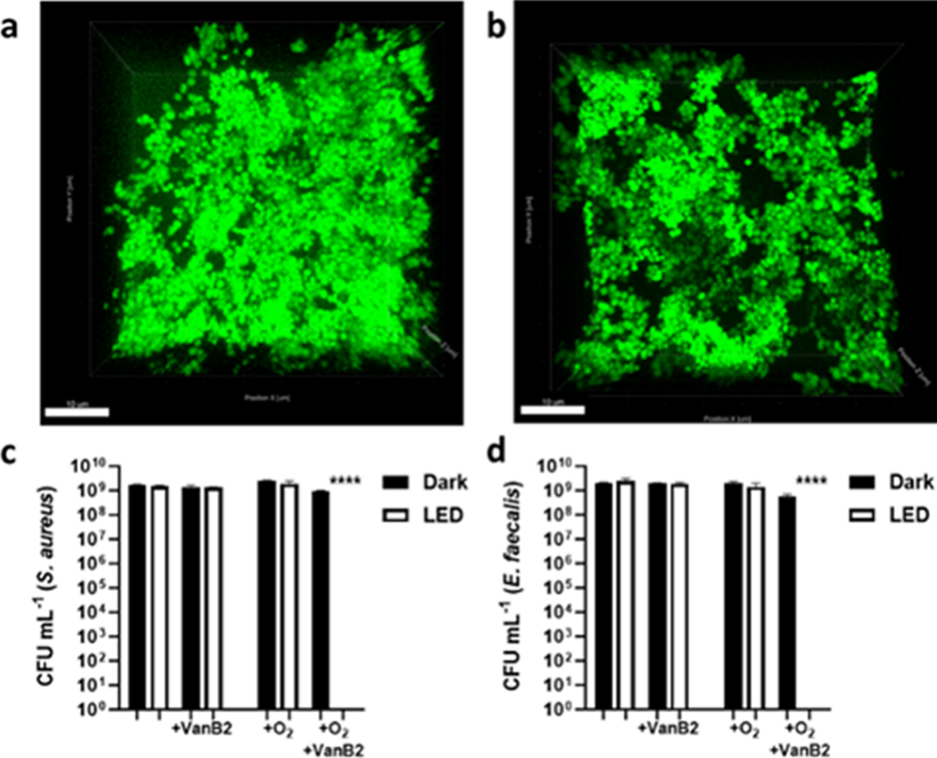
aPDT activity of VanB2 against bacterial biofilm. Representative maximum image projection (MIP) confocal images of (a) *S. aureus* and (b) *E. faecalis* biofilms counterstained with Syto9 (green). Scale bar shows 10 μm. Biofilm depths range 20–30 μm. Colony forming units (CFU) of (c) *S. aureus* and (d) *E. faecalis* following aPDT treatment with VanB2 (100 μM), with or without supplemental oxygen and illumination (white bars, LED: 455 nm, 30 mW/cm^2^, 60 min). Controls (black bars) were maintained in the dark. *n* = 3.

We established large, tightly packed biofilms of *S. aureus* and *E. faecalis*, with depths ranging from 20 to 30 μm, and 10^9^ CFU mL^–1^ (∼100-fold higher than the planktonic bacteria experiments) (Figure 4). Under the same experimental conditions as the planktonic bacteria, the biofilms were impervious to aPDT-mediated killing. However, to better tackle the impermeable and hypoxic nature of biofilm environments,^43^ we combined a higher **VanB2** concentration (100 μM) with supplemental oxygen and increased LED exposure (60 min), which fully eradicated both the *S. aureus* and vancomycin-resistant *E. faecalis* biofilm (Figure 4). A combination of aPDT with nanocarriers could make this a viable therapeutic system, even in hypoxic environments.^44^

Finally, bacteria also have a number of virulence factors that may protect them from exogenous ROS.^45^ In particular, *S. aureus* has a variety of defense mechanisms which are upregulated following oxidative burst, such as antioxidant enzymes and small molecules.^46^ We evaluated **VanB2** against a panel of *S. aureus* strains (including methicillin resistant strain MRSA USA300), which exhibited a range of staphyloxanthin pigmentation. Staphyloxanthin is a membrane-bound antioxidant carotenoid responsible for the yellow-orange appearance of many *S. aureus* strains. It is believed to offer protection against oxidative stress, including by impairing neutrophil ROS mediated killing through its ability to scavenge hydroxyl radicals, with strains lacking the pigment more easily killed.^47^ Here, aPDT-mediated killing with **VanB2** was shown to be independent of staphyloxanthin carotenoid levels (Figure 3e). These results show that **VanB2** remains an efficient phototherapeutic agent against AMR pathogens, including in the presence of high concentrations of ROS-scavenging species.

Together, the selective, rapid, and efficient nature of **VanB2** mediated aPDT against drug-resistant Gram-positive bacteria was demonstrated even at low concentrations, which was not achievable by the PS or antibiotic alone. The combined aPDT and secondary conventional antimicrobial activity of **VanB2** could provide an important alternative treatment for wound infections caused by *E. faecalis*.^48^

### Subcellular Imaging of VanB2-Treated Gram-Positive Bacteria

**Figure 5:**
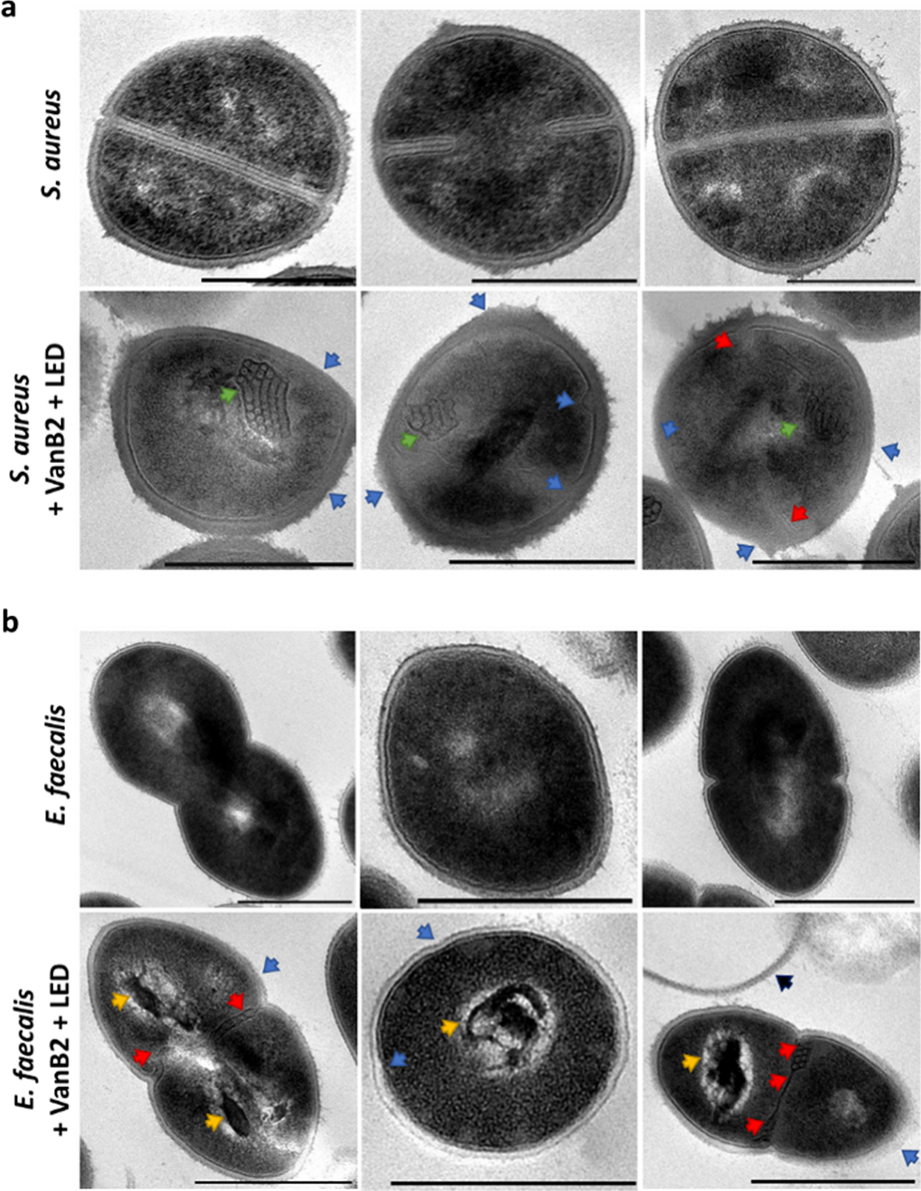
Transmission electron microscopy (TEM) of (a) *S. aureus* and (b) *E. faecalis*. Top panels: untreated controls, bottom panels: treated with VanB2 (5 μM) plus 20 min illumination (455 nm, 30 mW/cm^2^). Three representative images for each condition shown. Blue arrow indicates cell envelope damage; green arrow indicates mesosome-like structure; red arrow indicates irregular septum, yellow arrow indicates electron-dense areas, black arrow indicates a “ghost” cell wall. Scale bar = 500 nm.

Transmission electron microscopy (TEM) of *S. aureus* and *E. faecalis* following treatment with **VanB2** under blue light irradiation (along with untreated controls) was performed, with bacteria fixed immediately after PDT treatment. A number of morphological changes were observed across the **VanB2**-treated bacteria compared to controls (Figure 5). Post-treatment, *S. aureus* was shown to have a number of disruptions to the cell envelope and overall shape, including irregular cell envelope thickness, breaks in the plasma membrane, and rougher cell surfaces. Unlike the untreated bacteria, very few of the treated *S. aureus* had visible septum, and those which were visible were distorted. Furthermore, internal “mesosome-like” structures were observed only in PDT-treated cells. The association between mesosome-like structures and ROS^49^ or antibiotic (including vancomycin) damage within bacteria has been previously reported, and these were consistent features of our **VanB2**- and LED-treated *S. aureus*. TEM images of the **VanB2**-treated *E. faecalis* showed similar cell envelope damage and demonstrated major intracellular changes after treatment, with the formation of electron-dense aberrant structures and septum deformities. Occasional “ghost” cell envelope structures were also observed, indicating separation of the cell wall from the protoplast. Photoactivated **VanB2** is therefore able to elicit extracellular and intracellular cell damage through ^1^O_2_ diffusion across bacterial cell walls and plasma membranes.

### Off-Target Effects of VanB2 against Mammalian Cells

Minimizing off-target effects of aPDT against mammalian host cells is the key challenge of PS design strategies. We assessed membrane toxicity of **VanB2** and riboflavin (0–50 μM) using a hemolysis assay with primary human erythrocytes (Figure S16a) and cell viability against HaCaT keratinocyte skin cell-line (Figure S16b). No hemolysis was observed for **VanB2** at any concentration, even with illumination. This is in contrast to 60% erythrocyte lysis following LED activation of 50 μM riboflavin (Figure S16a). Riboflavin is known to accumulate within erythrocytes,^50^ and the lack of hemolytic activity for **VanB2** suggests it does not accumulate within these cells.

High concentrations (50 μM) of **VanB2** after irradiation reduced the viability of the HaCaT cells by 50%, with a comparable effect seen with 50 μM riboflavin and photoactivation. The blue-light activated riboflavin also caused ∼30% reduction in cellular viability at 5 μM, which was not observed for **VanB2** (Figure S16b). Since concentrations of **VanB2** of <1.25 μM were sufficient to induce complete Gram-positive bacterial killing, this shows that the lowest working concentrations of **VanB2** would have no negative impact on cellular viability. Importantly, the byproduct of the photolysis reaction lumichrome is also known to have no toxicity on human cells.^51^ These doses, as well as the higher concentration of **VanB2** required for complete eradication of the large bacterial biofilms (100 μM), also remain significantly lower than the 0.1% (2.66 mM) riboflavin solution used in clinic for the treatment of keratoconus by corneal cross-linking with UVA irradiation. Although further toxicity studies are warranted as a next step to translation, this indicates that **VanB2** could be a comparably safe option for biofilm treatment.^52^

## Conclusions

The novel vancomycin-riboflavin conjugate reported here (**VanB2**) was prepared on a gram scale in only two synthetic steps and revealed both selective photodynamic bacterial killing, potent bactericidal activity, and an unexpected drug-release behavior. Thanks to the triplet diradical formed upon excitation, we discovered for the first time that the flavinylethyl moiety behaves as a light-cleavable protecting group for vancomycin, with blue light irradiation triggering an oxygen-independent photochemical cascade leading to bond cleavage and drug release. In a biological context, this means that flavinyl groups can be used for light-mediated drug release, even in cells where no oxygen is present (e.g., anaerobic bacteria, tumor tissues, etc), which increases versatility. Therefore, in addition to their efficient photosensitizing character, flavins could therefore represent an entirely new family of “caging” compounds suitable for PDT and/or light-activated prodrug strategies. In the present antibacterial application, photodynamic treatment with **VanB2** was 100% efficient against Gram-positive pathogens even when used for short periods of time, and at submicromolar concentrations. Its activity was not affected by the presence of two types of resistance mechanisms, allowing the eradication of ESKAPE pathogens VRE and MRSA, and we were able to demonstrate efficacy against bacterial biofilms when supplying oxygen to the microenvironment. The conjugation strategy used here allows maximization of the therapeutic effect of the probe by concentrating the photodynamic killing with thousands of ^1^O_2_ molecules generated per PS molecule localized onto the target itself,^11^ thus avoiding off-target effects on mammalian cells. Thanks to the transformation of the riboflavin subunit into lumichrome during the light treatment, the absorption of the compound is significantly UV-shifted. This could also become a beneficial clinical attribute by preventing long-lasting sensitization of the treated area to visible light, which is a known source of painful side effects in conventional PDT treatment^53^ (i.e., internal “burning” due to the ongoing ROS generation, even in ambient light). Additionally, the conjugation of riboflavin onto the glycan moiety of vancomycin enhanced its antibacterial activity by a factor of ∼10 and even helped overcome vancomycin resistance in VRE bacteria. This increases the versatility of the **VanB2** probe, which overall proved to be highly potent both in the dark and under blue light, thus making it a promising, versatile alternative in the fight against infections.^54^ Future work will investigate the detailed scope and release mechanism of flavinylethyl light cleavable protecting groups.

## Methods

### Photophysical and Photochemical Studies

All photophysical studies were performed with freshly prepared solutions at room temperature (298 K) contained in standard 1 cm quartz cuvettes. Fluorescence quantum yields were measured according to literature procedures using fluorescein (Φ_f_ = 0.90 in NaOH 0.1 M, λ_exc_ = 474 nm) as reference (see ESI).

All irradiation experiments (^1^O_2_, ROS, and photocleavage) were performed in an optical cage cube (Thorlabs LC6W) equipped with a mounted LED array (Thorlabs LIU470A, 4.0 mW/cm^2^ at 470 nm). For ^1^O_2_ and ROS generation experiments, air-saturated solutions containing the probe (10 μM) and the appropriate sensor (100 μM ABMDMA, or 10 μM DHR123, respectively) were irradiated and monitored over time by UV–vis and fluorescence spectroscopy, respectively. Singlet oxygen quantum yields were obtained using solutions of (−)-Riboflavin (Φ_Δ_ = 0.54 in water, Φ_Δ_ = 0.48 in MeOH) irradiated in identical conditions as reference (see ESI).

For photocleavage experiments, solutions of the probe (5 μM) in PBS (2 mL) were irradiated for 20 min. The reaction was monitored over time by measuring the UV–vis absorption spectra of the solution, and at each time point, aliquots (50 μL) were collected and eluted on an RP-HPLC coupled to a mass spectrometer in ESI mode (*m*/*z* = 100–1000 in positive mode). Quantification of the conversion rate by RP-HPLC-MS allowed to determine the uncaging quantum yield Φ_u_ of the reaction by comparison with a potassium ferrioxalate actinometry reference (see ESI).

^1^H NMR monitoring of the uncaging reaction was performed by irradiating solutions of the probe (100 μM) in DMSO-*d*_6_ (0.6 mL) in a glass NMR tube placed directly into the optical cage cube via a pierced cover plate (Thorlabs LB6C). ^1^H NMR spectra (600 MHz) were recorded at regular intervals to monitor the photolysis.

### Bacterial Strains and Culture Conditions

Bacterial strains utilized within this study were *Escherichia coli* (ATCC 25922), *Enterococcus faecalis* (ATCC 51299), and *Staphylococcus aureus* (SH1000, Xen29, USA300, Newman, ATCC 25923, ATCC 8325-4). Unless otherwise stated, *S. aureus* ATCC 25923 was utilized in the study. Bacteria were sourced from the local culture collection at the University of Edinburgh. Single bacterial colonies were selected from Luria–Bertani Broth (LB) agar plates (Sigma-Aldrich, L7025) and inoculated into LB broth, grown overnight at 37 °C in under constant motion (Sciquip Incushake Midi).

Planktonic Bacteria: overnight cultures were adjusted to OD_595_ 0.1 (Biotech Photometer) and incubated until mid-log phase (OD_595_ 0.4–0.8) under the same conditions. Bacteria concentrations were readjusted to the final concentration of OD_595_ 0.1 in sterile saline (0.9% NaCl, Baxter). The bacteria were washed 3 times with sterile saline, centrifuged for 1 min at 10,600 × *g* (Sigma 1–14 Microfuge), and resuspended in sterile saline for aPDT and imaging experiments.

Bacterial biofilms: overnight cultures of *S. aureus* 25923 or *E. faecalis* were diluted to an OD_595_ 0.01 in Tryptic Soy Broth (TSB) broth (Sigma-Aldrich) in a 48 flat-bottomed well plate (Corning, Costar 3548). The plates were incubated at 37 °C without shaking for 24 h. Following this, the TSB media was carefully removed, and biofilms were gently washed (by pipetting) in 0.9% NaCl, ready for further experimentation.

### aPDT Treatment with VanB2

#### Planktonic Bacteria

aPDT experiments were performed with bacterial strains listed and prepared as described above with **VanB2**; vancomycin and riboflavin served as probe controls. Unless otherwise stated, compounds were utilized at a final concentration of 5 μM and incubated with the prepared bacteria in a total volume of 300 μL for 10 min in the dark at room temperature. Where required, bacteria were washed by centrifugation at 10,600 x *g,* followed by the replacement of the supernatant with 0.9% NaCl sterile saline. Subsequently, the bacteria requiring illumination were transferred into appropriate wells of a 96-well plate and placed into the LED device described in ESI. The samples were illuminated by the LED (455 nm) for up to 20 min, as indicated within the text providing an irradiance of up to 36 J cm^–2^. Control treatments were kept in the dark. Experiments were repeated independently 3 times.

#### Bacterial Biofilms

**VanB2** (100 μM, 300 μL) was added to biofilms and illuminated with the LED device for 60 min. Where required, oxygen was bubbled into the biofilm media at a flow rate of 1 L min^–1^ via placement of tubing (Nipro Safetouch winged needle sets, 19Gx 3/4, with the needles removed) at the media meniscus. Oxygen was delivered throughout the illumination period (and equivalent duration in the dark controls). Experiments were repeated independently 3 times.

### Enumerating aPDT Bacterial Killing

#### Planktonic Bacteria

Following aPDT treatment (or dark controls), 10-fold serial dilutions in sterile saline were prepared for bacteria colony forming unit (CFU) plating. Each dilution was plated onto LB agar in triplicate and incubated overnight in a static incubator at 37 °C. Colony forming units (CFUs) were counted the following day and presented as average CFU mL^–1^.

#### Biofilm Bacteria

Following treatment, biofilms were transferred to Precellys tubes and homogenized to disperse biofilms for CFU plating. For the quantification of PDT bacterial killing, serial dilutions in sterile saline were prepared. Each dilution was plated onto LB agar in triplicate and incubated overnight in a static incubator at 37 °C. CFU were counted the following day and presented as average CFU mL^–1^.

Further details of methodology are presented in the Electronic Supporting Information.

## Supporting Information Available

Complete experimental procedures and methods for chemistry, photophysics, and biology experiments, including supplementary figures, schemes, and tables, and chemical characterization (NMR, MS, UV–vis, fluorescence spectra, HPLC chromatograms, and NMR spectra). The data presented in this study is openly available in Edinburgh DataShare at 10.7488/ds/3852 (PDF)
