# Supplementary material for: Riboflavin-Vancomycin Conjugate Enables Simultaneous Antibiotic Photo-Release and Photodynamic Killing against Resistant Gram-Positive Pathogens
Source: JACS Au. 2023 Oct 24;3(11):3014–23. doi: 10.1021/jacsau.3c00369 (PMC10685426; doi:10.1021/jacsau.3c00369)
Supplement: Supplementary file 1 — au3c00369_si_001.pdf [file au3c00369_si_001.pdf]

Riboflavin-Vancomycin Conjugate Enables Simultaneous Antibiotic Photo-Release and Photodynamic Killing against Resistant Gram-Positive Pathogens

Bethany Mills,[#*2] Alex Kiang, [#2] Syam Mohan P. C. Mohanan,[2] Mark Bradley,[1] and Maxime Klausen*[1]

1 Prof. M. Bradley, Dr. M. Klausen
EaStCHEM School of Chemistry, University of Edinburgh, David Brewster Road, EH9 3FJ Edinburgh, UK.
E-mail: mklausen@ed.ac.uk, m.klausen@imperial.ac.uk

2 Dr. B. Mills, A. Kiang, Dr. S. M. P. C. Mohanan
Translational Healthcare Technologies group, Centre for Inflammation Research, Queen's Medical Research Institute, University of Edinburgh, 47 Little France Crescent, Edinburgh EH16 4TJ, UK.
E-mail: beth.mills@ed.ac.uk
[#] Authors contributed equally

SUPPORTING INFORMATION

Table of content

I. Supporting Figures, Schemes and Tables S2
II. Materials and Methods S15
1. Synthesis and chemical characterisation S15
2. Photophysical experiments S15
3. Singlet oxygen generation, ROS generation and photolysis experiments S16
4. Dark stability S18
5. Biology S18
III. Synthesis and characterization S23
IV. NMR, MS, and HPLC Data S25
V. References S30

I. Supporting Figures, Schemes and Tables

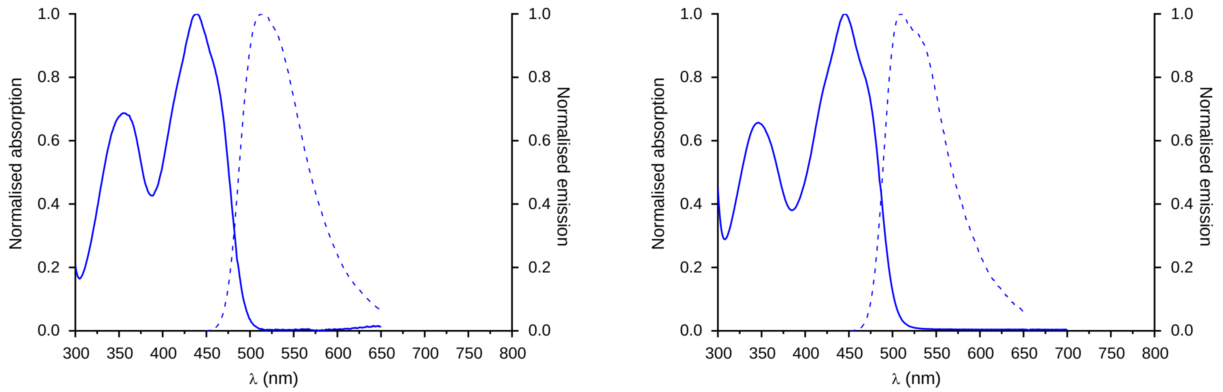

Figure S1. Normalised absorption and normalised emission spectra of **VanB2** in MeOH (left) and DMSO (right).

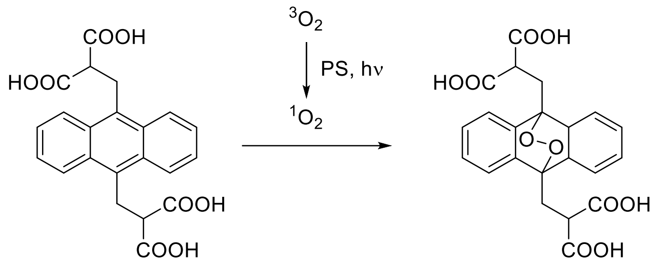

Scheme S1. Photo-oxidation of AMBDMA by singlet oxygen occurring upon excitation of a photosensitizer.

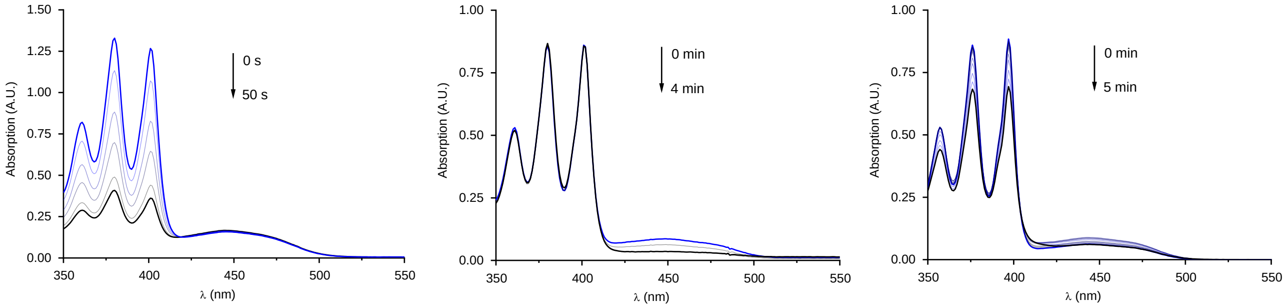

Figure S2. Evolution of the absorption of solutions containing the $^1O_2$ sensor ABMDMA and **Riboflavin** in PBS (left), DMSO (middle) and MeOH (right) upon excitation at 470 nm.

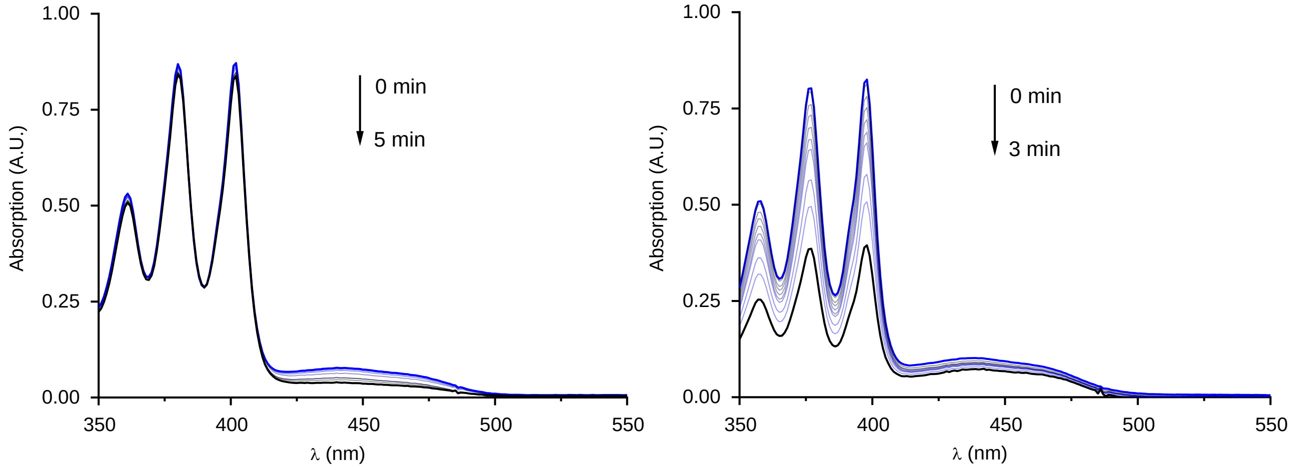

Figure S3. Evolution of the absorption of solutions containing the $^1O_2$ sensor ABMDMA and **VanB2** in DMSO (left) and MeOH (right) upon excitation at 470 nm.

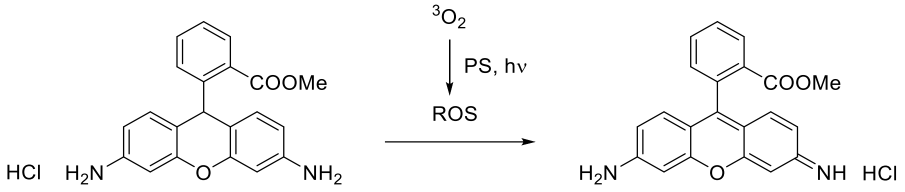

Scheme S2. Light mediated-oxidation of Dihydrorhodamine 123 (DHR123) into Rhodamine 123 by ROS generated upon excitation of a photosensitizer.

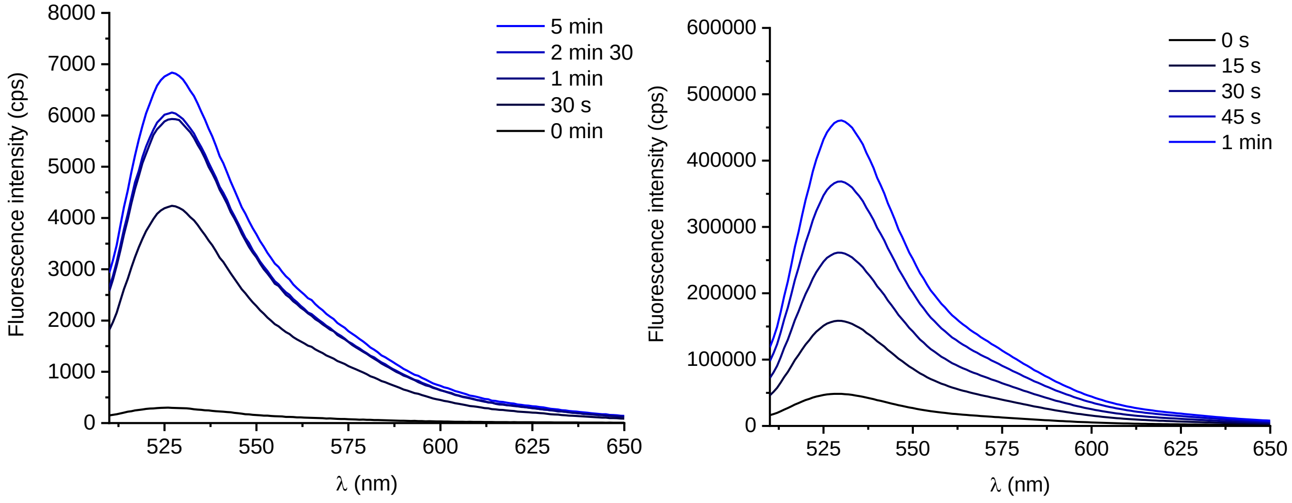

Figure S4. Evolution of the fluorescence of solutions containing the ROS sensor DHR123 (10 μM) and Riboflavin (10 μM) in PBS (left) and MeOH (right) upon excitation at 470 nm.

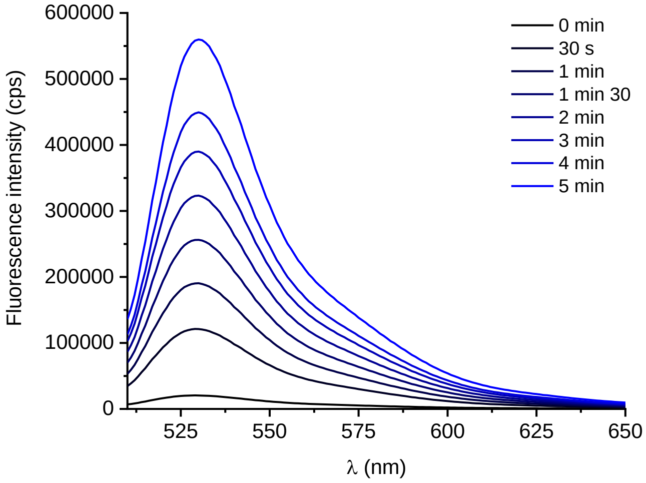

Figure S5. Evolution of the fluorescence of solutions containing the ROS sensor DHR123 (10 μM) and **VanB2** (10 μM) in MeOH upon excitation at 470 nm.

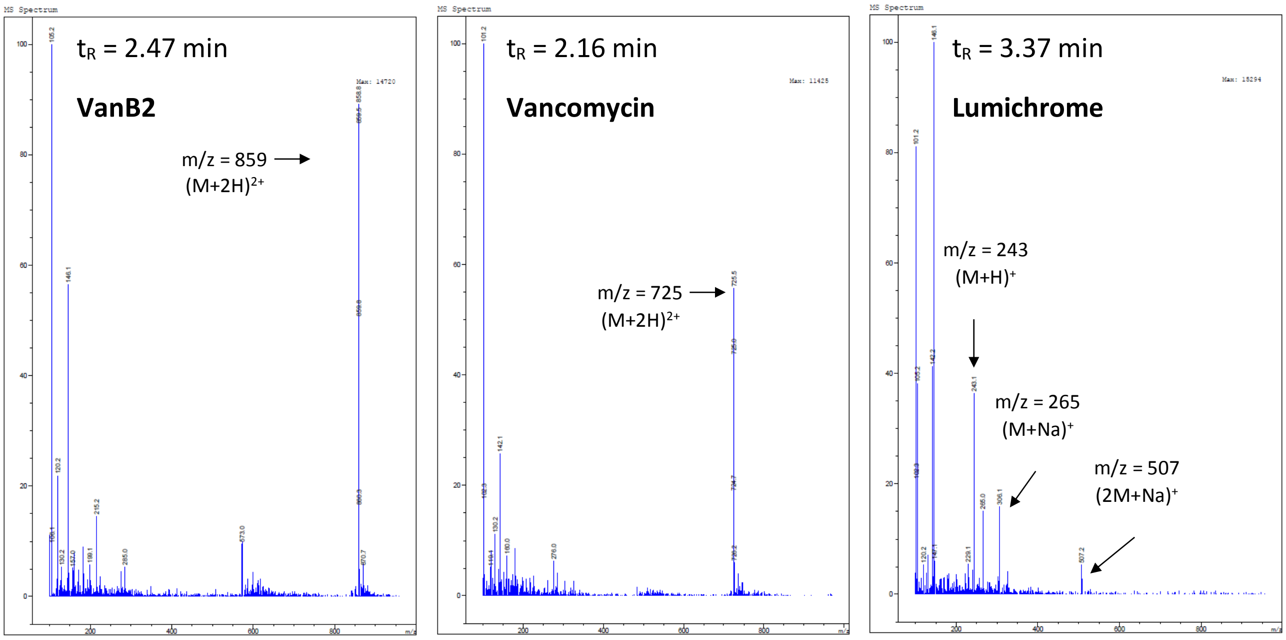

Figure S6. LC-MS spectra (ESI positive mode, m/z = 100-1000) of the peaks identified after 10 min of irradiation of **VanB2** at 470 nm in PBS.

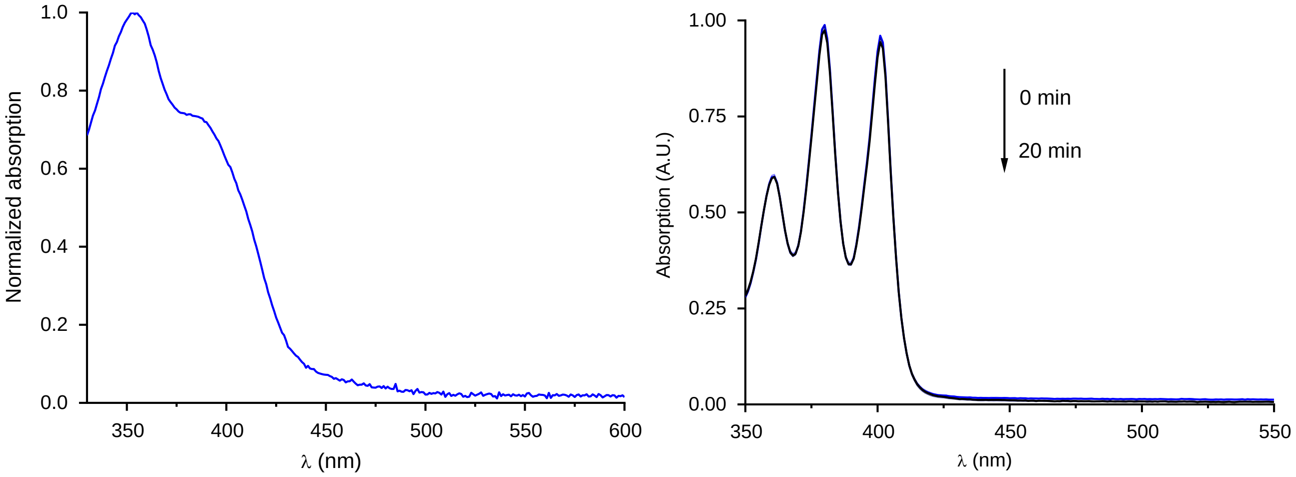

Figure S7. Normalised absorption spectrum of lumichrome in PBS (left), and control experiment showing no evolution in the absorption of solutions containing the $^1O_2$ sensor ABMDMA (100 µM) and lumichrome (10 µM) in PBS upon excitation at 470 nm (right).

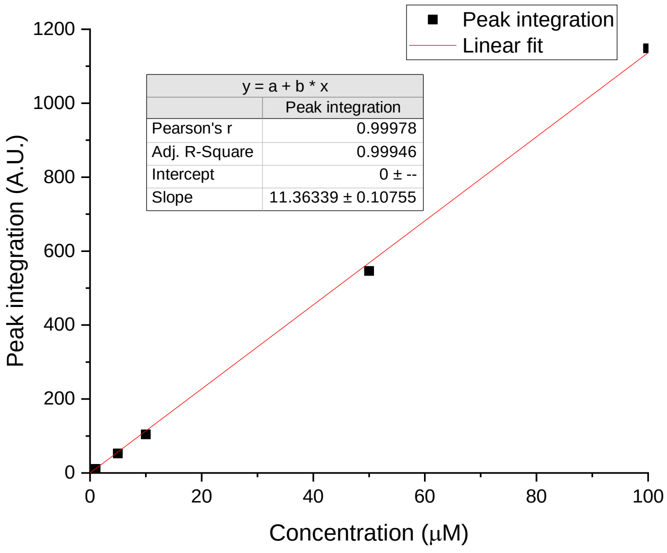

Figure S8. External calibration curve showing the area of HPLC peaks of vancomycin samples as a function of concentration.

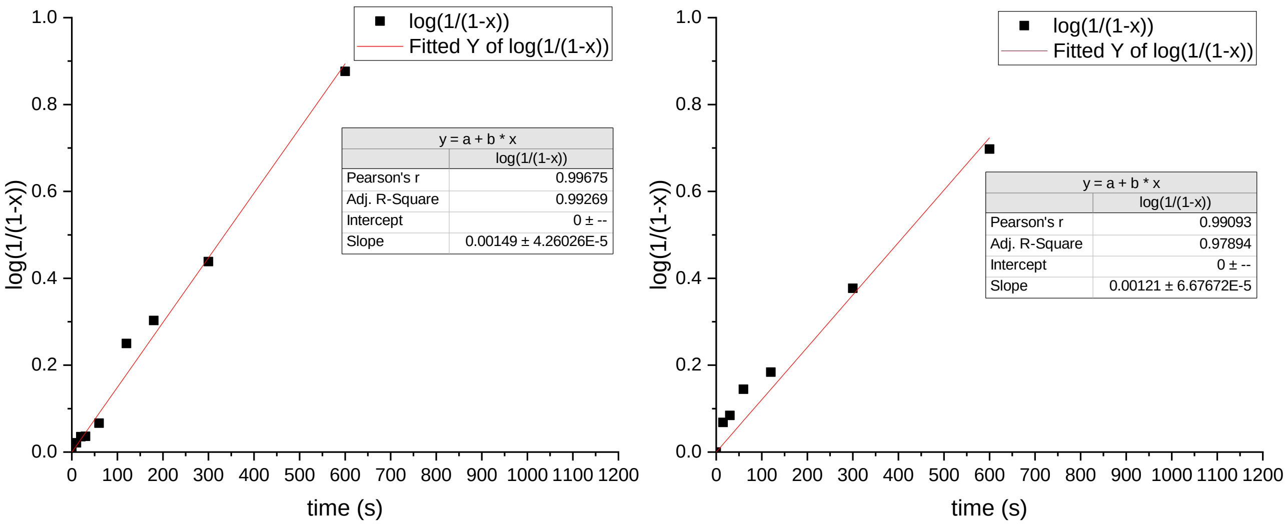

Figure S9. Kinetics of the photolysis reaction occurring upon irradiation at 470 nm of VanB2 (5 μM) in air-saturated PBS (left) and in N_2-saturated PBS (right); derived from HPLC analysis.

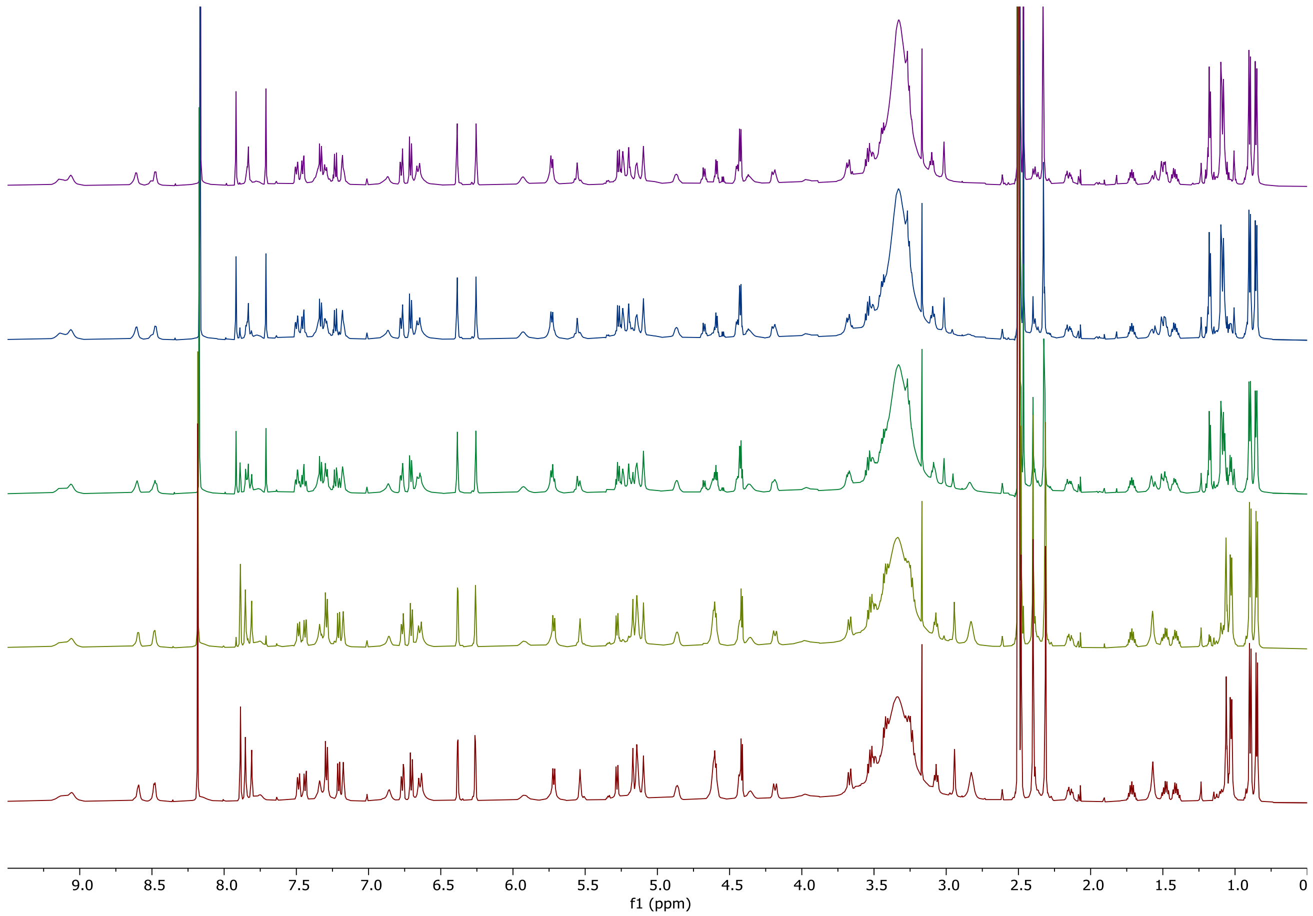

Figure S10. Evolution of the ^{1}H NMR spectrum of **VanB2** upon irradiation at 470 nm in DMSO-d_6 after 0, 1, 5, 7 and 10 min (bottom to top).

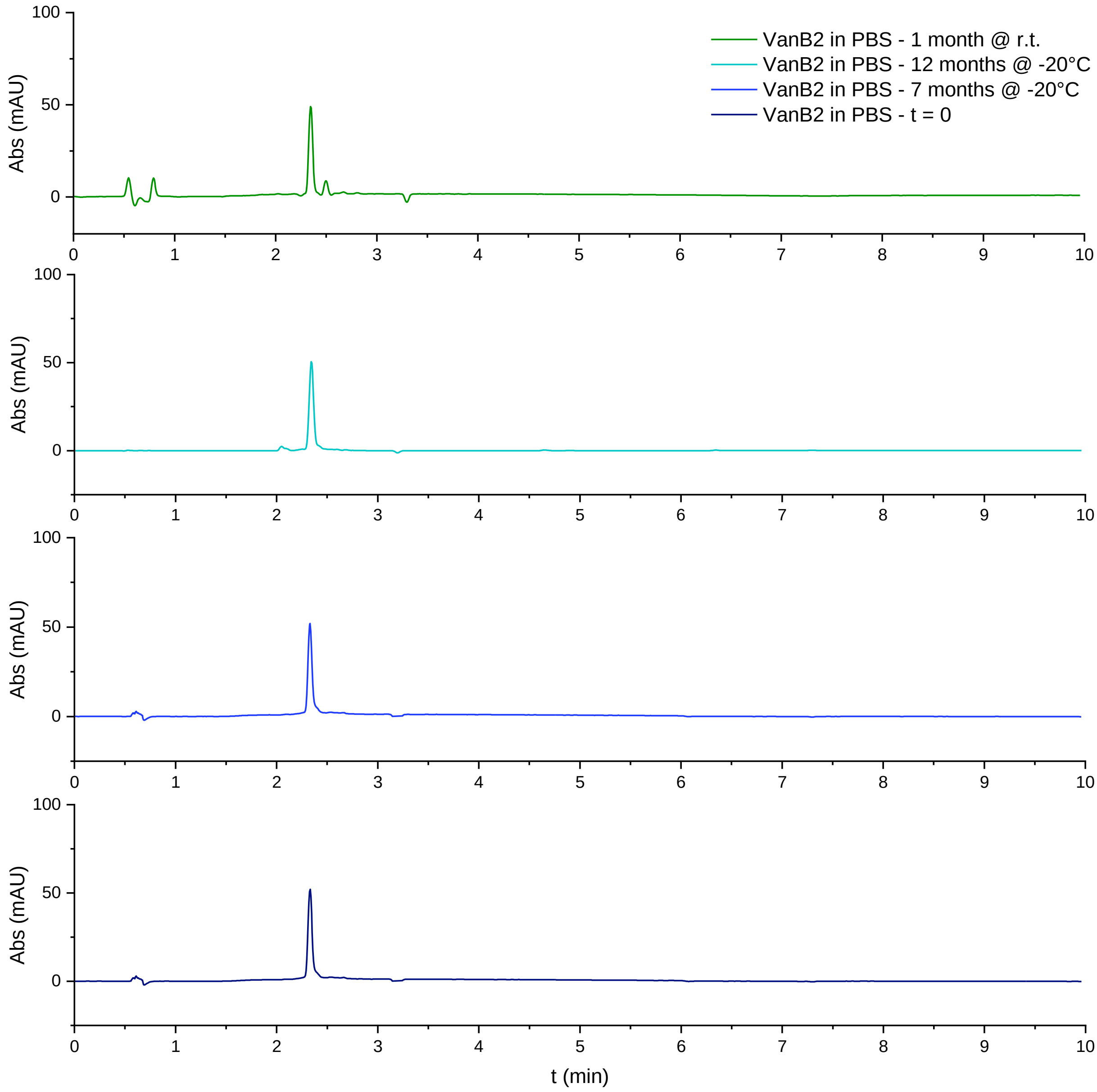

Figure S11. HPLC traces (elution with method A, UV-Vis detection at 440 nm, see materials & methods section) of a solution of **VanB2** in PBS upon storage at -20 °C over 12 months, or storage at room temperature over 1 month.

a

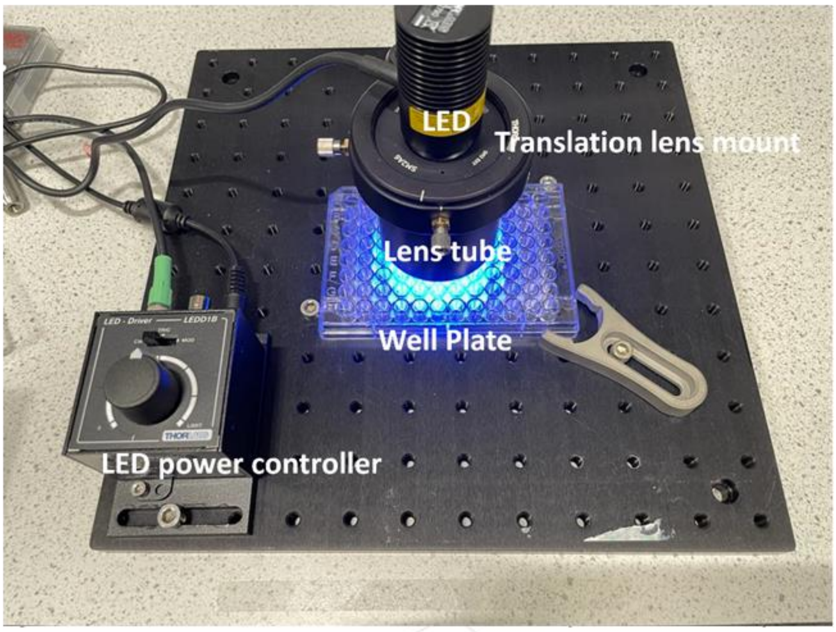

b

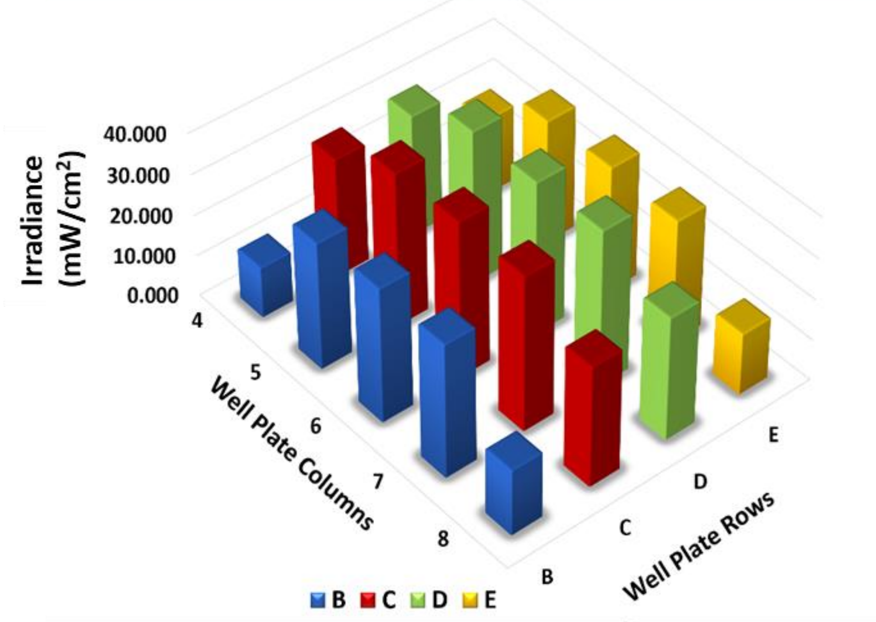

Figure S12. PDT irradiation set-up. (a) Photograph of bespoke LED set-up for 96-well plate illumination. **(b)** Measured average irradiance per well. Samples were randomly allocated to positions B5-B7, C5 -C7, and D5-D7 for irradiation. N = 3.

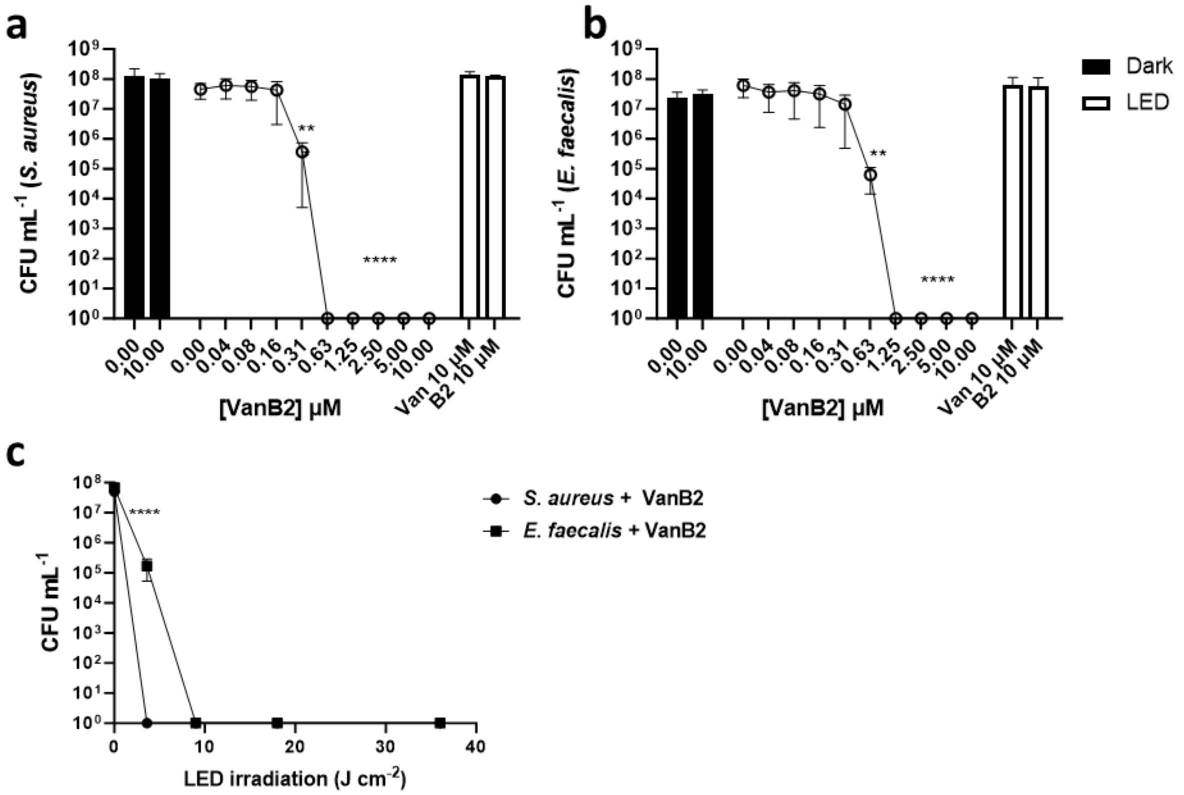

Figure S13. Limit of aPDT activity of VanB2 against *S. aureus* and *E. faecalis*. Colony forming units (CFU) of (a) *S. aureus* and (b) *E. faecalis* were recorded following treatment with increasing concentrations of **VanB2** or with 10 µM vancomycin (van) or riboflavin (B2). Compounds were added for 10 min prior to removal of the excess compound followed by 20 min illumination (455 nm, 30 mW cm^{-2}). Controls were maintained in the dark with or without compound. (c) CFU of *S. aureus* (circles) and *E. faecalis* (squares) labelled with 5 µM **VanB2** following LED illumination (36 J cm^{-2} is equivalent 20 min illumination (455 nm, 30 mW cm^{-2}). Error bars show s.e.m., *n* = 3.

Table S1. Traditional vancomycin susceptibility MIC evaluation of *S. aureus* and *E. faecalis*.

	[Vancomycin] μg mL^{-1}						
	**0**	**5** (3 μM)	**10** (7 μM)	**20** (14 μM)	**30** (21 μM)	**50** (34 μM)	**100** (69 μM)
S. aureus **(ATCC 25923)**	Growth	No growth	No growth	No growth	No growth	No growth	No growth
E. faecalis **(ATCC 51299)**	Growth	Growth	Growth	Growth	Growth	Growth	No growth

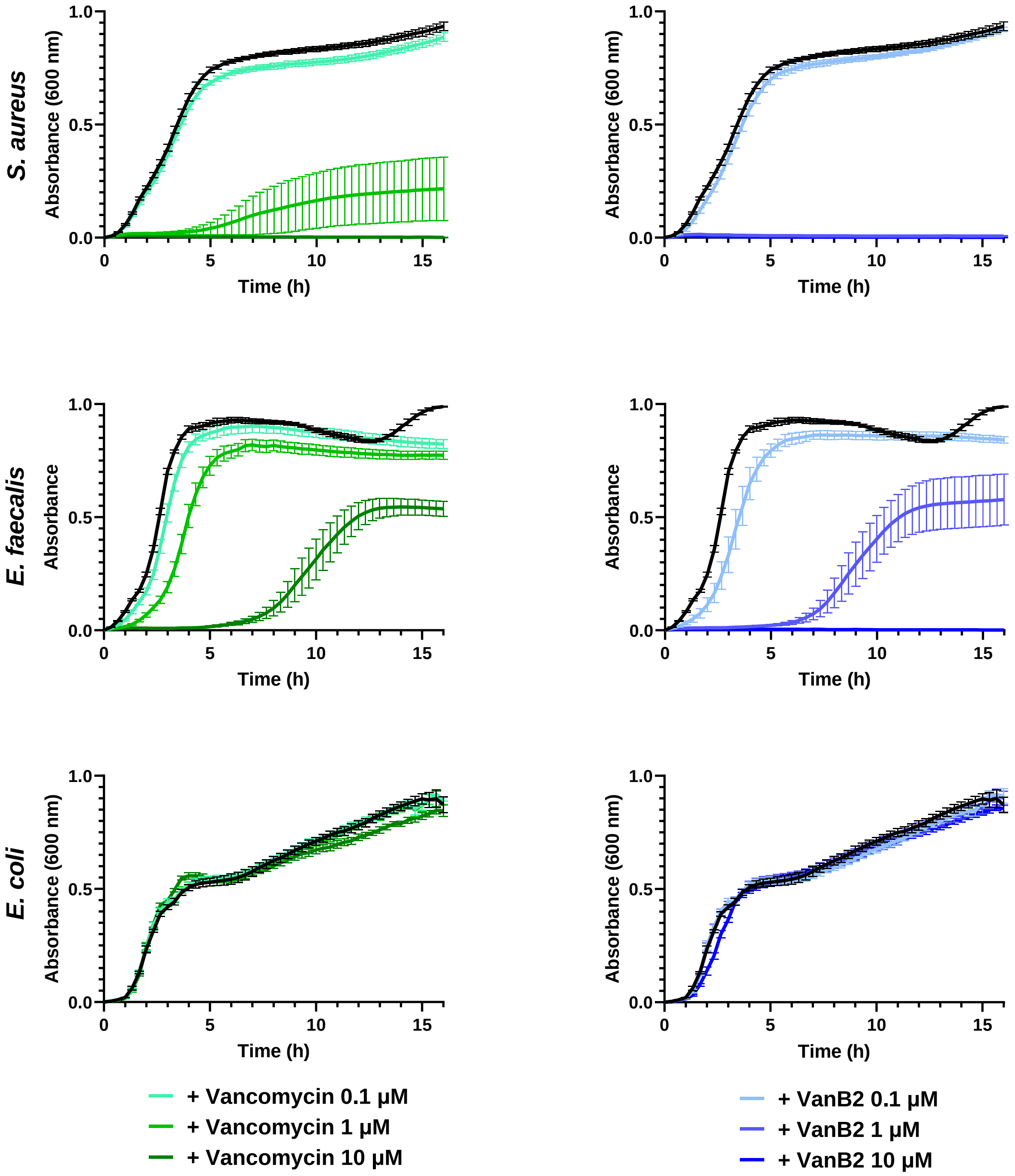

Figure S14. Growth rate of bacteria in the presence of vancomycin (green) or VanB2 (blue). Black curves show compound-free bacterial growth rates. Error bars show s.e.m. n = 3.

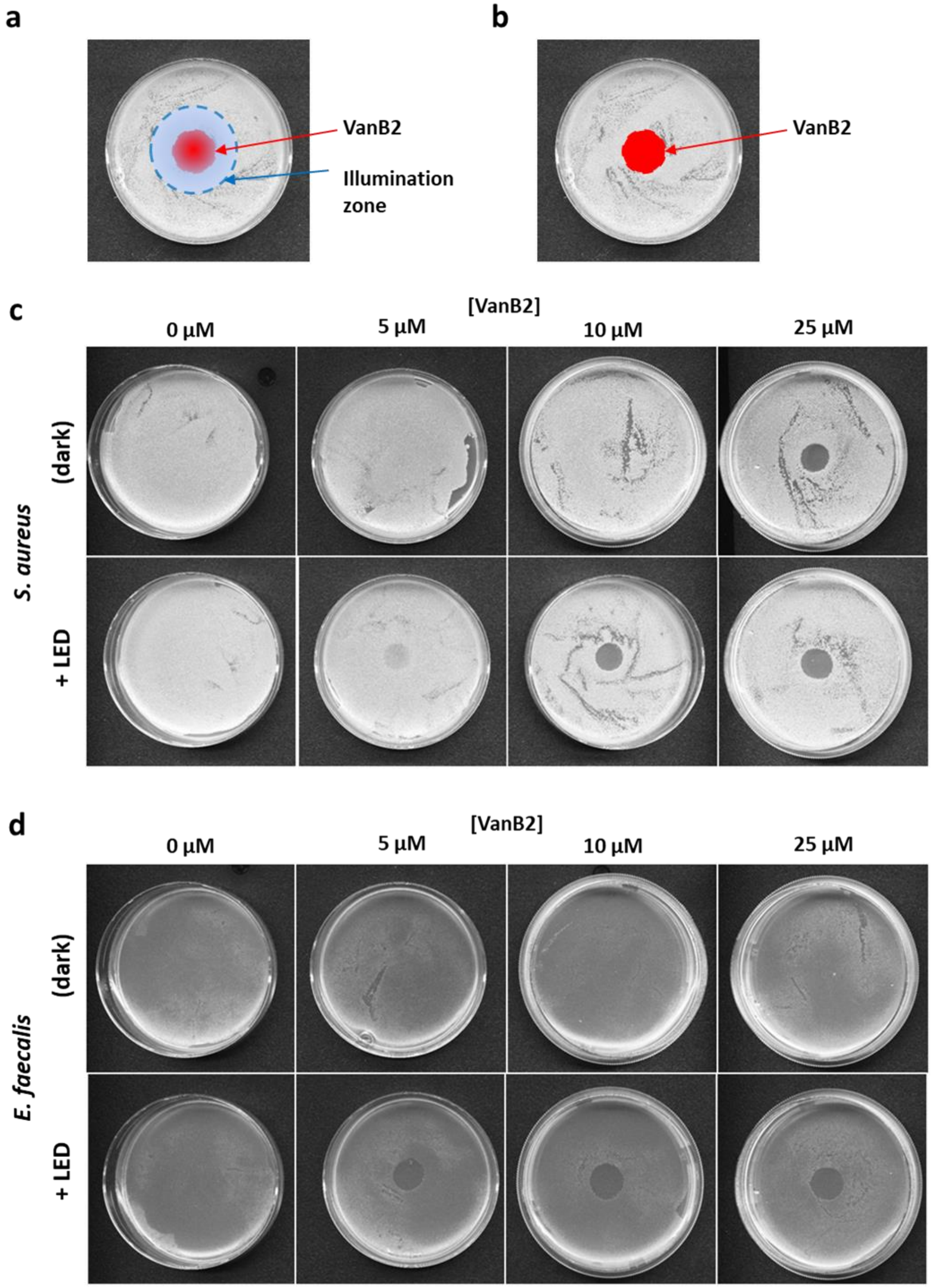

Figure S15. VanB2 aPDT killing of *S. aureus* and *E. faecalis* lawns. Lawns of bacteria were spread, VanB2 was added to the centre of the plate and **(a)** illuminated or **(b)** maintained in the dark. Schematics show area of VanB2 delivery and illumination. Representative images of **(c)** *S. aureus* and **(d)** *E. faecalis* growth (and growth inhibition zones) on agar following illumination (and dark controls) with increasing concentrations of VanB2.

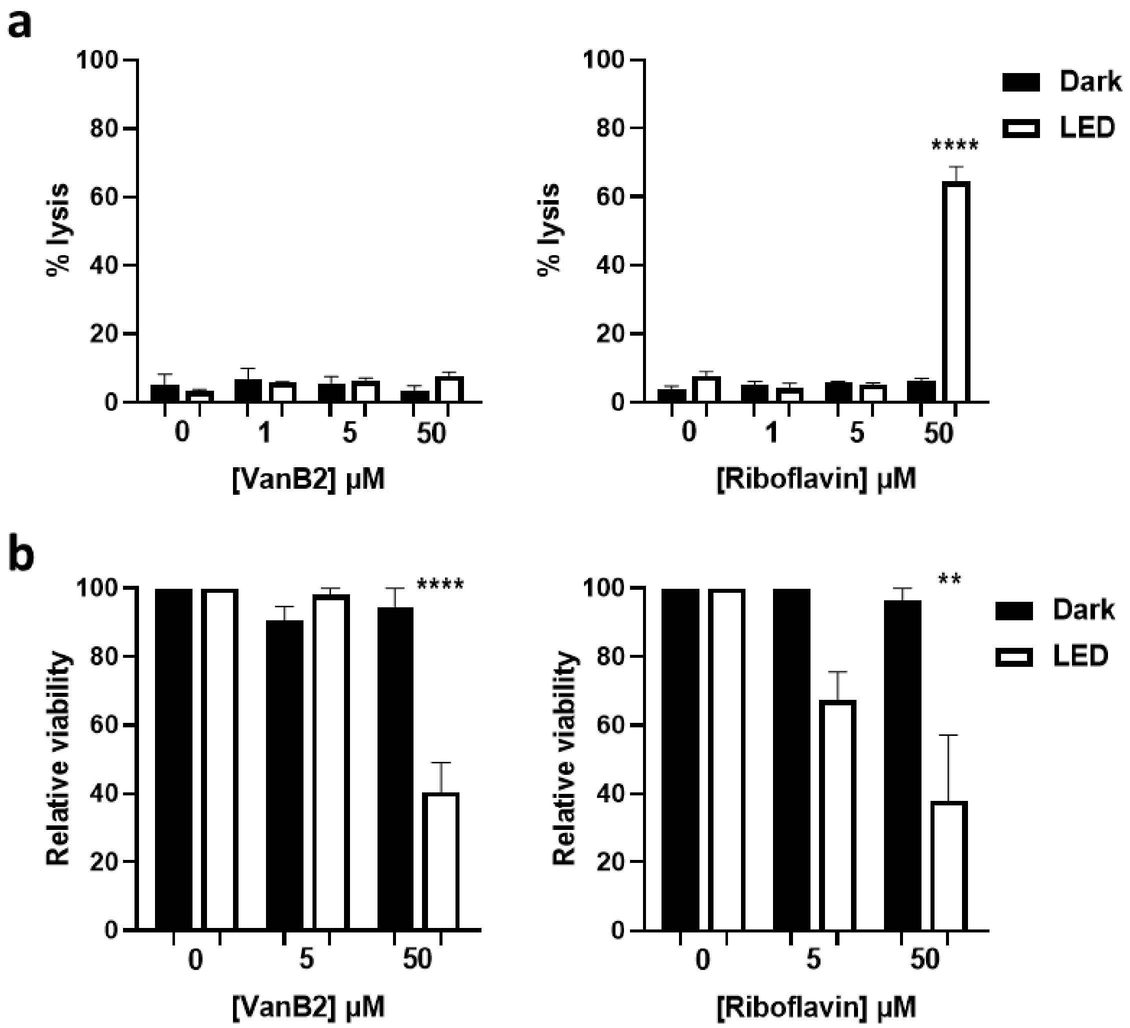

Figure S16. Off-target effects of VanB2 against mammalian cells. (a) Haemolysis of human erythrocytes following PDT with **VanB2** or riboflavin (0-50 μM), measured by absorbance at 455 nm. **(b)** Relative viability of HaCaT keratinocyte cell-line following PDT with **VanB2** or riboflavin (0-50 μM) was determined by WST-1 assay and measured absorbance at 455 nm. For all, the compounds were added for 10 min followed by removal of excess compound, and 20 min illumination (455 nm, 30 mW/cm^2). Error bars show s.e.m., analysed one-way ANOVA with comparison to no-compound control: ****$P < 0.0001$, **$P = 0.0011$. $n = 3$.

II. Materials and Methods

1. Synthesis and chemical characterisation

All air and moisture sensitive manipulations were carried out using standard techniques, with oven-dried reaction vessels, anhydrous solvents, and under nitrogen atmosphere. Extraction and column chromatography solvents were purchased in anhydrous form, and used as received. All reagents were purchased from Fisher Scientific, Aldrich or Fluorochem and used without further purification unless indicated otherwise. Lumichrome (> 90 %) was purchased from Cayman Chemicals and used without further purification. Thin layer chromatography (TLC) was performed on Merck silica gel 60 F254 aluminum plaques, and column chromatography was performed on Macherey-Nagel silica gel 60 (40-63 µm).

^{1}H and ^{13}C spectra were recorded on a Bruker AVA 500 spectrometer at 500 MHz and 126 MHz, or on a Bruker AVA 600 spectrometer at 600 MHz and 151 MHz respectively. Shifts (δ) are given in parts per million with respect to the non-deuterated solvent residual peak for ^{1}H spectra, and relative to the deuterated carbon resonance for ^{13}C and coupling constants (*J*) are given in Hertz.

Analytical reverse-phase high-performance liquid chromatography (RP-HPLC) was performed on an Agilent 1100 system equipped with a Kinetex XB-C18 column (50 × 4.6 mm, 5 µm) with a flow rate of 1 mL/min. Samples were eluted either with a gradient of H_2O/MeCN 95/5 to 5/95 (method A) or H_2O/MeOH 95/5 to 5/95 (method B), all buffered with 0.1% formic acid, over 6 min, then holding at 95% for 3 min, followed by elution at 5% MeCN. Detection was performed with a multiple wavelength detector (MWD) at 254, 282, and 440 nm, and by an evaporative light scattering (ELSD) detector.

Reverse phase flash chromatography was performed on a Biotage® Isolera One purification system using Biotage® SNAP Ultra C18 columns.

Low resolution electrospray ionization mass spectrometry (ESI-MS) analyses were carried out on an Agilent Technologies LC/MSD Series 1100 quadrupole mass spectrometer (QMS) in ESI mode. HR-MS were obtained by the Mass Spectrometry department of the University of Edinburgh and were performed on a Finnigan MAT 900 XLP high resolution double-focusing mass spectrometer. MALDI spectra were acquired on a Bruker Ultraflextreme MALDI TOF/TOF with a matrix solution of sinapinic acid (10 mg/mL) in H_2O/CH_3CN/TFA (50/50/0.1).

2. Photophysical experiments

All photophysical studies were performed with freshly prepared air-equilibrated solutions at room temperature (298 K). UV/Vis absorption spectra of ~10^{-5} M solutions were recorded on an Agilent 8453 spectrophotometer. Steady-state fluorescence measurements were performed on dilute solutions (ca. 10^{-6} M,

optical density ≤ 0.1) contained in standard l = 1 cm quartz cuvettes using a Shimadzu RF-6000 spectrofluorometer. The emission spectra were corrected for the wavelength-sensitivity of the detection unit, obtained, for each compound, under excitation at the wavelength of the absorption maximum. Fluorescence quantum yields of these dilute chromophore solutions were measured according to literature procedures[1,2] using Fluorescein (FLSCN, Φ_f = 0.9 in NaOH 0.1 M, λ_{exc} = 474 nm) as reference.[3] The emission quantum yield values derived from these measurements were calculated with the following equation taking into account the refractive index n, the absorbance A, and the integral of the emission $I_f\ (\lambda_{exc}, \lambda_f)$ of the novel sample (superscript *S*) relative to the reference (superscript *ref*):

$$\Phi_f^S = \Phi_f^{ref} \times \left(\frac{n^S}{n^{ref}}\right)^2 \times \frac{1 - 10^{-A^{ref}(\lambda_{exc})}}{1 - 10^{-A^S(\lambda_{exc})}} \times \frac{\int_0^\infty I_f^S(\lambda_{exc}, \lambda_f)\, d\lambda_f}{\int_0^\infty I_f^{ref}(\lambda_{exc}, \lambda_f)\, d\lambda_f}$$

3. Singlet oxygen generation, ROS generation and photolysis experiments

3.1 Singlet oxygen generation

The relative singlet oxygen quantum yield (Φ_Δ) of the new probes was determined according to the relative method described in literature,[4] using ABMDMA as singlet oxygen trap. Air saturated solutions of the probe (10 μM) and ABMDMA (100 μM) in the desired solvent were irradiated in an optical cage cube (Thorlabs LC6W) equipped with a mounted LED array (Thorlabs LIU470A, 4.0 mW/cm^2 at 470 nm). During the course of the irradiation, the absorbance at 380 nm of the sample was monitored over time in order to follow the photo-oxidation reaction occurring between the ABMDMA sensor and the $^1O_2$ generated (scheme S1). The absorption spectra of the samples irradiated (1 to 30 min – depending on the efficacy of the photosensitizing agent) – are shown in Figures 1, S2, and S3. The kinetics of the photo-oxidation reaction for the novel probe was then compared with an appropriate reference ((-)-**Riboflavin,** Φ_Δ = 54% in water,[5] Φ_Δ = 48% in MeOH[6]) irradiated under identical experimental conditions. The quantum yields were then calculated according to the following equation:

$$\Phi_\Delta^S = \Phi_\Delta^{ref} \times \left(\frac{m^S}{m^{ref}}\right) \times \frac{1 - 10^{-A^{ref}(\lambda_{irr})}}{1 - 10^{-A^S(\lambda_{irr})}}$$

Where the superscripts *S* and *ref* represent respectively the measured PS sample and the known reference, m is the slope of the decrease in absorbance (A-A$_0$) of the $^1O_2$ sensor at 380 nm over time, and $1 - 10^{-A\ (\lambda_{irr})}$ is a correction factor taking into account the difference in optical density of the samples at the irradiation wavelength (470 nm).

Note: because of the 1:10 ratio of probe:ABMDMA, the change in absorption in the UV related to the photodegradation of the flavin unit and concomitant formation of Lumichrome was considered negligible.

3.2 Reactive oxygen species generation

The ability to generate ROS (other than singlet oxygen) upon irradiation of the new photosensitizing agents was investigated using dihydrorhodamine 123 (DHR 123) as a sensor. Air saturated solutions of the PS (10 μM) and DHR123 (10 μM) in water or MeOH were irradiated in an optical cage cube (Thorlabs LC6W) equipped with a mounted LED array (Thorlabs LIU470A, 4.0 mW/cm^2 at 470 nm). During the course of the irradiation, the fluorescence intensity of the samples at 528 nm (λ_{exc} = 500 nm) was monitored over time in order to follow the light mediated oxidation of DHR123 into the fluorescent rhodamine 123 (scheme S2). The emission spectra of the samples (irradiated for 1 to 30 min – depending on the efficacy of the photosensitizing agent) – are shown in Figures 1, S4 andS5.

3.3 Photo-release of vancomycin

The photolysis of **VanB2** was performed by irradiating solutions of the probe (5 μM, 2 mL) in PBS in an optical cage cube (Thorlabs LC6W) equipped with a mounted LED array (Thorlabs LIU470A, 4.0 mW/cm^2 at 470 nm) for up to 20 min. When performed in aerobic conditions, the cuvette was sealed with a septum and kept under a continuous flow of nitrogen via a needle. Aliquots (50 μL) of the photolysis reaction were taken at regular time points, and eluted (method A, see below) on an RP-HPLC coupled to a mass spectrometer in ESI mode scanning the range m/z = 100-1000 in positive mode. During the course of the irradiation, the peak related to **VanB2** (2.47 min) decreased, while peaks related to vancomycin (2.16 min) and lumichrome (3.37 min) appeared. The peaks were identified thanks to the MS spectra recorded at their retention time (Figure S6). The conversion rate (x) was determined by integration of the HPLC peaks, and verified a first order kinetics law (Figure S8).

The "uncaging" quantum yield Φ_u of **VanB2** was determined using the equation $\Phi_u = (I\sigma_\lambda t_{90\%})^{-1}$, where I is the irradiation intensity expressed in einstein·cm^2·s^{-1}, σ_λ is the decadic extinction coefficient at the excitation wavelength (i.e. $10^3 \times \varepsilon_\lambda$) in cm^2·mol^{-1}, and $t_{90\%}$ is the irradiation time (in seconds) corresponding to 90% conversion of the photolysis reaction.[7]

The total irradiation intensity I at 470 nm was measured using the well-described chemical actinometry method.[8,9] Briefly, a solution of potassium ferrioxalate (solution 1, V_1 = 2 mL, 0.15 M) in H_2SO_4 (0.05 M) was irradiated at 470 nm in conditions identical to **VanB2** (i.e. standard cuvette, l = 1 cm optical path, in the abovementionned optical cage cube) for 5 s. An identical solution was kept in the dark as control. Each solution was then mixed with a 0.1% solution of 1,10-phenanthroline dihydrate (solution 2, V_2 = 0.33 mL) in a $NaOAc\cdot3H_2O/H_2SO_4$ aqueous buffer. The absorbance of each sample was measured at 510 nm. The number of

moles of Fe^{2+} ions generated per unit time of irradiation (n^{Fe2+} in mol·s^{-1}), was determined using the following equation:

$$n^{Fe2+} = \frac{(V_1 + V_2) \times \Delta A_{510}}{t \times l \times \varepsilon_{510}}$$

Where ΔA_{510} is the change in absorbance of the Fe^{2+}-phen complex at 510 nm before and after irradiation (ΔA_{510} = 0.93), ε_{510} is the molar absorptivity of the Fe^{2+}-phen complex at 510 nm ($\varepsilon_{510} = 1.1 \times 10^4$ $M^{-1}cm^{-1}$, according to literature[9]), l is the optical path length (cm), t is the time of irradiation of the potassium ferrioxalate solution (t = 5 s), and $V_1 + V_2$ is the total volume of solution ($V_1 + V_2 = 2.33 \times 10^{-3}$ L).

The irradiation intensity *I* of the 470 nm light supply was then further calculated using the following equation:

$$I = \frac{n^{Fe2+}}{\Phi_{Fe2+} \times (1 - 10^{-A_{470}})}$$

Where Φ_{Fe2+} is the quantum yield of production of ferrous ions by photoreduction of ferrioxalate at the excitation wavelength. The value reported for photoreduction of a 0.15 M ferrioxalate solution at 468 nm was used here (Φ_{Fe2+} = 0.93)[9]. The correction factor $(1 - 10^{-A_{470}})$, where A_{470} is the absorbance of the ferrioxalate solution at the irradiation wavelength (A_{470} = 0.50), is used to account for the high concentration of potassium ferrioxalate in actinometry experiments at 470 nm.

Using the absorption spectrum of **VanB2** in PBS (Figure 1) and the first order kinetic plots (Figure S8) allowed to determine the σ_{470} and $t_{90\%}$ parameters respectively ($\varepsilon_{470} = 9.0 \times 10^3$ M^{-1} cm^{-1}; $t_{90\%}$ = 671 s), and therefore the Φ_u of **VanB2**.

4. Dark stability

The dark stability of a solution of **VanB2** (PBS, 100 μM) stored in a freezer (-20 °C) or at room temperature (20 °C) was assessed over time by HPLC, eluting with method A (see below). No significant change in the HPLC trace were observed under such conditions (Figure S11).

5. Biology

5.1 Preparation of solutions

Concentrated stock solutions of the following compounds were prepared from powder in phosphate buffered saline (PBS, Sigma-Aldrich L7275-100TAB): **VanB2** (100 μM) and Riboflavin (100 μM, Alfa Aesar, A11764). Vancomycin (Vancomycin Hydrochloride (Sigma-Aldrich, SBR00001-10ML) was diluted to 100 μM in PBS. All compounds were stored at -20 ˚C in the dark.

5.2 Bacterial strains and culture conditions

Bacterial strains utilised within this study were *Escherichia coli* (ATCC 25922), *Enterococcus faecalis* (ATCC 51299), and *Staphylococcus aureus* (SH1000, Xen29, USA300, Newman, ATCC 25923, ATCC 8325-4). Unless otherwise stated, *S. aureus* ATCC 25923 was utilised in the study. Bacteria were sourced from the local culture collection at the University of Edinburgh. Single bacterial colonies were selected from Luria Bertani Broth (LB) agar plates (Sigma-Aldrich, L7025) and inoculated into LB broth, grown overnight at 37 °C in under constant motion (Sciquip Incushake Midi).

Planktonic Bacteria: Overnight cultures were adjusted to OD_{595} 0.1 (Biotech Photometer) and incubated until mid-log phase (OD_{595} 0.4-0.8) under the same conditions. Bacteria concentrations were readjusted to the final concentration of OD_{595} 0.1 in sterile saline (0.9 % NaCl, Baxter). The bacteria were washed 3 times with sterile saline, and 1 min centrifugation at 10,600 x *g* (Sigma 1-14 Microfuge) and re-suspended in sterile saline for aPDT and imaging experiments.

Bacterial biofilms: Overnight cultures of *S. aureus* 25923 or *E. faecalis* were diluted to an OD_{595} 0.01 in Tryptic Soy Broth (TSB) broth (Sigma-Aldrich) in 48 flat-bottomed well plate (Corning, Costar 3548). The plates were incubated at 37 °C without shaking for 24 h. Following which, the TSB media was carefully removed, and biofilms were gently washed (by pipetting) in 0.9% NaCl, ready for further experimentation.

5.3 Determining vancomycin susceptibility and resistance

The vancomycin susceptibility/resistance of *E. faecalis* and *S. aureus* was determined by growing the strains on solid LB supplemented with increasing concentrations of vancomycin (0 – 100 µg mL^{-1}). The agar plates were inoculated with bacteria grown overnight as described above and spread to form a lawn (100 µL). All plates were incubated at 37 ˚C, 5 % CO_2 for 24 h. Plates were then visually inspected for bacterial growth. Experiments were repeated independently 3 times.

5.4 aPDT equipment set-up

Prepared bacteria were irradiated for aPDT in 96-well plates (Corning Costar™ 3596). The irradiation of light to the 96 well-plate was achieved using a light emitting diode (LED) with centre wavelength of 455 nm (M455L3, Thorlabs Ltd) and full width half maximum of 18 nm. Homogeneous illumination over the desired wells was produced using an aspheric condenser lens (ACL50832U, Thorlabs Ltd) with a diameter of 50.8 mm and focal length of 32 mm. A downward configuration system was developed for the illumination as shown in Supplementary Fig. xS12). The output power of the LED was adjusted by a current controller connected to the LED using an M8 four pin connector. The LED was attached to one side of the translating lens mount (LM2XY/M, Thorlabs Ltd) using an adaptor (SM2A6, Thorlabs Ltd), the lens tube and lens were connected on the other side of the translation mount. The well-plate was housed at a distance of 2 cm below the illumination setup. An optical power meter and power head (PM100D and S130C, Thorlabs Ltd) were used to measure the

optical power to each well in the well-plate. The aperture of the power head was fixed at 0.6 cm diameter, which was equivalent to the diameter of a well in the well plate. The power mapping was performed by moving the sensor head on each well to measure the optical power. The irradiance (mW/cm^2) was calculated as power (mW) / area of exposure.

The measured power for each well at the centre of the light exposure (wells B5-B7, C5 - C7, and D5 - D7) was 10.5 ± 0.5 mW and the corresponding irradiance was 30.0 ± 0.5 mW/cm^2, average and standard deviation from 3 independent repeats (Supplementary Fig. S12). These wells were selected for experimentation, with samples assigned randomly to each well.

5.5 aPDT treatment with VanB2

5.5.1. Planktonic bacteria: aPDT experiments were performed with bacterial strains listed and prepared as described above with **VanB2**; vancomycin and riboflavin serving as probe controls. Unless otherwise stated, compounds were utilised at a final concentration of 5 µM, and incubated with the prepared bacteria in a total volume of 300 µL for 10 min in the dark at room temperature. Where required, bacteria were washed by centrifugation at 10,600 x *g,* followed by replacement of the supernatant with 0.9% NaCl sterile saline. Subsequently the bacteria requiring illumination were transferred into appropriate wells of a 96-well plate, and placed into the device outlined above. The samples were illuminated by the LED (455 nm) for up to 20 min, as indicated within the text providing an irradiance of up to 36 J cm^{-2}. Control treatments were kept in the dark. Experiments were repeated independently 3 times.

5.5.2. Bacterial lawns: planktonic bacteria were prepared as described above and resuspended to a final OD_{595} 0.01 on LB broth. 100 µL of bacterial suspension was spread evenly across LB agar plates. The plates were allowed to air-dry, and 10 µL of VanB2 (0-25 µM) was pipetted onto the centre of the plate. Plates were maintained in the dark at room temperature until illuminated with the 455 nm LED (20 min, 30 mW/cm^2) . The area of illumination was greater than the area of VanB2 on the plate. Plates were incubated at 37 °C for 16h and examined for colony growth. Experiments were repeated independently 3 times.

5.5.3. Bacterial biofilms: 300 µL VanB2 (100 µM) was added to biofilms and illuminated with the LED device for 60 min. Where required, oxygen was bubbled into the biofilm media at a flow rate of 1 L min^{-1} via placement of tubing (Nipro Safetouch winged needle sets, 19Gx ¾, with the needles removed) at the media meniscus. Oxygen was delivered throughout the illumination period (and equivalent duration in the dark controls). Experiments were repeated independently 3 times.

5.6 Enumerating aPDT bacterial killing

5.6.1. Planktonic bacteria: Following aPDT treatment (or dark controls), ten-fold serial dilutions in sterile saline were prepared for bacteria colony forming unit (CFU) plating. Each dilution was plated onto LB

agar in triplicate and incubated overnight in a static incubator at 37 °C. Colony forming units (CFUs) were counted the following day and presented as average CFU mL^{-1}.

5.6.2. Biofilm bacteria: Following treatment, biofilms were transferred to Precellys tubes and homogenized to disperse biofilms for CFU plating. For the quantification of PDT bacterial killing, serial dilutions in sterile saline were prepared. Each dilution was plated onto LB agar in triplicate and incubated overnight in a static incubator at 37 °C. CFU were counted the following day and presented as average CFU mL^{-1}.

5.7 Confocal laser scanning microscopy (CLSM) of bacterial biofilm

Biofilms were grown directly in in 8-well imaging chamber slides (Ibidi 80826) as outlined above, Prior to imaging, biofilms were gently washed in sterile saline and counterstained with 5 µM Syto9 Green Fluorescent Nucelic Acid Stain (Thermo Fisher) as per manufactures instructions. Confocal microscopy was performed on a Leica SP8 (HC PL APO CS2 63x 1.40 oil, HyD detectors) using 488 nm excitation. A minimum of 3 fields of view were captured per condition and repeated independently three times. Images were processed using the Leica Application Suite X (LAS X) software.

5.8 Transmission Electron Microscopy (TEM)

Following aPDT treatment, *S. aureus* and *E. faecalis* were prepared for TEM. Samples were centrifuged and fixed in 3 % glutaraldehyde in 0.1 M Sodium Cacodylate buffer, pH 7.3, for 2 h then washed in three 10 min changes of 0.1 M Sodium Cacodylate. Specimens were then post-fixed in 1 % Osmium Tetroxide in 0.1 M Sodium Cacodylate for 45 min, then washed in three 10 min changes of 0.1 M Sodium Cacodylate buffer. These samples were then dehydrated in 50 %, 70 %, 90 % and 100 % ethanol (3 times) for 15 min each, then in two 10 min changes in Propylene Oxide. Samples were then embedded in TAAB 812 resin. Sections, 1 µm thick, were cut on a Leica Ultracut ultramicrotome, stained with Toluidine Blue, and viewed in a light microscope to select suitable areas for investigation. Ultrathin sections, 60 nm thick were cut from selected areas, stained in Uranyl Acetate and Lead Citrate then viewed in a JEOL JEM-1400 Plus TEM. Representative images were collected on a GATAN OneView camera, with a minimum of 10 fields of view captured. Images were processed (cropped as necessary and scale bar added) with Fiji Image J.

5.9 Bacterial growth inhibition assay

Bacteria were grown and prepared as described above. 180 µL of an OD_{595} 0.1 of each bacterium in Muller Hinton broth was added into wells of a 96-well plate (Corning) with each compound (vancomycin or

VanB2 (0-10 µM) added to a total volume of 200 µL. Bacterial growth over time was measured by microplate reader (BioTek Synergy H1 multi-mode reader), with absorbance measured at 600 nm. Data were collected in duplicate from three independent repeats.

5.10 Haemolysis Assay

Blood was collected from healthy human volunteers following ethical approval (AMREC 20-HV-069) and informed consent. Erythrocytes were isolated and resuspended in 0.9 % NaCl to the original volume. Erythrocytes were diluted to 0.7 % (v/v) in PBS, washed and aliquoted. **VanB2** and riboflavin were added at increasing final concentrations (0 µM – 50 µM). These were maintained in the dark or illuminated (455 nm LED: 10 mW, 20 min). This was followed by incubation at 37 ˚C for 40 min after which the samples were centrifuged at 400 x *g* for 10 min and supernatant absorbance (415 nm) was measured by plate-reader (BioTek™ Synergy™ HTX Multi-Mode Microplate Reader). % lysis was determined from interpolation from a standard curve prepared from lysed (probe sonicated) erythrocytes for each experimental repeat and normalised for intrinsic absorbance from **VanB2** and riboflavin at each concentration tested. The experiment was performed independently 3 times.

5.11 HaCaT WST-1 cell viability assay

Human keratinocyte cell line HaCaT (Cell Line Service; Article 300493) were grown in Dulbecco's Modified Eagle Medium (DMEM, Gibco ™ 11965084) containing fetal bovine serum (5 %, Gibco ™ A5209401), L-glutamine (0.5 %, Gibco ™ 25030-081) and Pen-Strep (0.5 %, Gibco™ 15140-122) at 37 ˚C, 5 % CO_2. Once 80 % confluent, cells (4×10^5 cells mL^{-1}) were seeded into 48 well plates (Corning) and incubated at 37 ˚C, 5 % CO_2 for 24 h. **VanB2** or riboflavin (0 - 50 µM) were added to the cells followed by incubation (10 min, dark). The cells then underwent illumination (455 nm LED: 10 mW, 20 min) or were maintained in the dark. All cells were then incubated in the dark at 37 °C for the remainder of 2 h. Cell Proliferation Reagent WST-1 (Roche 5015944001) was added (50 µL, volume equal to 10 % of total well volume) and incubated at 37 °C 5% CO_2 for 4 h. Absorbance of well supernatant (100 µL in duplicate) was measured at 450 nm by plate reader (BioTek™ Synergy™ HTX Multi-Mode Microplate Reader). % viability was calculated by interpolating data via simple linear regression on a line fitted from 100 % viability (untreated HaCaT cells) to 0 % viability (70% methanol treated HaCaT cells). Experimentation was repeated independently 3 times.

5.12 Statistical analysis

Throughout, data were plotted and analysed with GraphPad Prims 9. For determining statistical relationships among conditions, CFU counts were analysed using one-way ANOVA on log-transformed data.

III. Synthesis and characterization

10-(2-oxoethyl)-7,8-dimethylisoallozazine (1)[10]

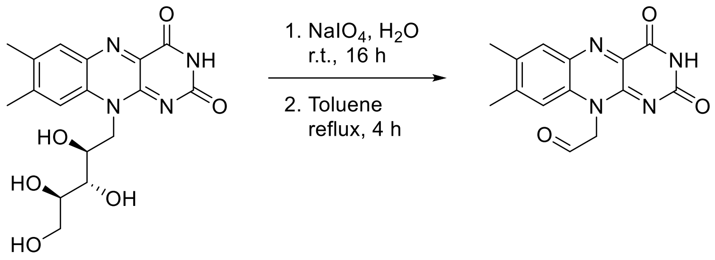

To a stirred suspension of riboflavin (5.0 g, 13.3 mmol, 1.0 eq.) in water (200 mL) was added sodium periodate (8.0 g, 37.2 mmol, 2.8 eq.). The mixture was stirred at room temperature overnight, then the suspension was filtered. The collected precipitate was washed with water, methanol, and diethyl ether successively, and dried in vacuum to give the intermediate geminal diol (containing about 15% of the aldehyde).

^{1}H NMR (601 MHz, DMSO-*d*$_6$) δ 11.31 (s, 1H), 7.91 (s, 1H), 7.87 (s, 1H), 6.28 (d, *J* = 5.1 Hz, 2H), 5.34 – 5.30 (m, 1H), 4.57 (d, *J* = 5.5 Hz, 2H), 2.49 (s, 3H), 2.39 (s, 3H). **HPLC (UV detection, 282 nm):** 2.65 min (Ge10ACN).

The resulting powder was refluxed in toluene for 4 h, then the mixture was cooled down to r.t. The precipitate was collected by filteration, washed with diethyl ether and air dried to give the expected aldehyde as a light orange powder (3.2 g, **85%**).

^{1}H NMR (601 MHz, DMSO-*d*$_6$) δ 9.73 (s, 1H), 7.94 (s, 1H), 7.70 (s, 1H), 5.64 (s, 2H), 2.46 (s, 3H), 2.40 (s, 3H). **^{13}C NMR (126 MHz, DMSO-*d*$_6$) δ** 195.3, 159.7, 155.3, 150.1, 146.9, 137.1, 136.1, 133.5, 131.0, 116.3, 53.8, 20.5, 18.8. **HRMS (ESI):** m/z = 285.0977, calcd for $C_{14}H_{13}N_4O_3$ [M+H]$^+$: 285.09822. **HPLC (Method B, UV detection, 254 nm):** 2.59 min. **Rf:** 0.65 (CH_2Cl_2:MeOH, 95:5), UV, DNP stained.

Preparation of VanB2

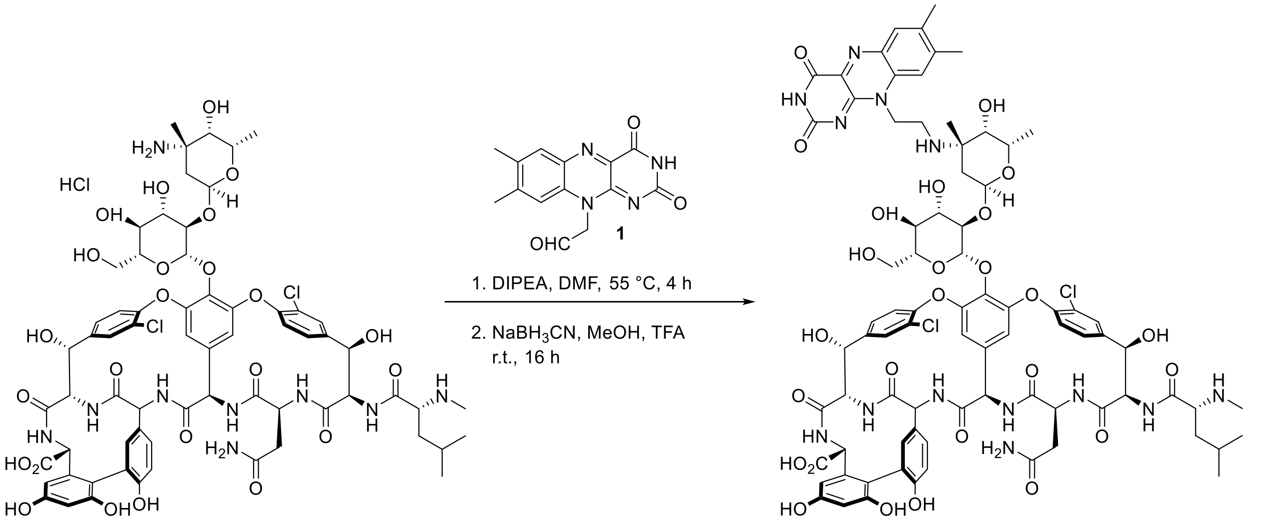

Vancomycin hydrochloride (2.0 g, 1.35 mmol, 1.0 equiv.), riboflavin aldehyde **1** (765 mg, 2.69 mmol, 2 equiv.), and DIPEA (0.61 mL, 3.50 mmol, 2.6 equiv.) were stirred in DMF (60 mL) under a N_2 atmosphere at 55 °C for 4 h. The solution was then cooled down to room temperature and a solution of sodium cyanoborohydride (161 mg, 2.56 mmol, 1.9 equiv.) in MeOH (15 mL) was added to the mixture. TFA (0.61 mL, 8.08 mmol, 6 equiv.) was finally added and the reaction was stirred at room temperature for 16 h. The reacting mixture was then concentrated to dryness, and dissolved in H_2O/MeCN (40 mL, 9/1 v/v, with 0.1% formic acid), then centrifuged. The supernatant was purified by Isolera® RP flash chromatography (C18, 60 g cartridge) eluting with H_2O/MeCN, 0.1% formic acid (95/5 to 40/60). Fractions were combined and evaporated to give **VanB2** as a bright yellow solid (1.04 g, **45%**).

^{1}H NMR (500 MHz, DMSO-*d*$_6$) δ 11.29 (s, 1H), 9.07 (s, 2H), 8.61 – 8.56 (m, 1H), 8.45 (d, *J* = 5.9 Hz, 1H), 8.24 (s, 2H), 8.16 (s, 1H), 7.87 (d, *J* = 13.0 Hz, 2H), 7.82 (d, *J* = 1.9 Hz, 1H), 7.51 – 7.46 (m, 1H), 7.46 – 7.41 (m, 1H), 7.34 (s, 1H), 7.29 (d, *J* = 8.4 Hz, 2H), 7.23 – 7.16 (m, 2H), 6.85 (s, 1H), 6.76 (dd, *J* = 8.4, 2.0 Hz, 1H), 6.70 (d, *J* = 8.5 Hz, 1H), 6.63 (d, *J* = 11.8 Hz, 2H), 6.38 (d, *J* = 2.2 Hz, 1H), 6.30 (s, 1H), 5.72 (d, *J* = 8.0 Hz, 1H), 5.53 (s, 1H), 5.28 (d, *J* = 7.7 Hz, 1H), 5.17 (d, *J* = 2.0 Hz, 1H), 5.16 – 5.12 (m, 2H), 5.10 (s, 1H), 4.87 (d, *J* = 8.0 Hz, 1H), 4.61 (p, *J* = 6.5 Hz, 3H), 4.42 (dd, *J* = 11.3, 5.5 Hz, 2H), 4.36 (s, 1H), 4.18 (d, *J* = 11.7 Hz, 1H), 3.67 (d, *J* = 10.8 Hz, 1H), 3.51 (dq, *J* = 11.0, 6.4 Hz, 2H), 3.42 (t, *J* = 8.7 Hz, 1H), 3.25 (p, *J* = 10.0 Hz, 2H), 3.10 (t, *J* = 7.2 Hz, 1H), 2.96 (s, 1H), 2.84 (s, 2H), 2.48 (s, 3H), 2.40 (s, 3H), 2.32 (s, 3H), 2.14 (dd, *J* = 15.8, 5.6 Hz, 1H), 1.71 (dp, *J* = 13.5, 6.7 Hz, 1H), 1.59 (s, 2H), 1.49 (dt, *J* = 13.7, 6.8 Hz, 1H), 1.42 (dd, *J* = 13.7, 7.0 Hz, 1H), 1.23 (s, 0H), 1.16 – 1.07 (m, 1H), 1.07 (s, 3H), 1.03 (d, *J* = 6.3 Hz, 3H), 0.89 (d, *J* = 6.6 Hz, 3H), 0.85 (d, *J* = 6.5 Hz, 3H). **^{13}C NMR (126 MHz, DMSO-*d*$_6$) δ** 174.1, 172.9, 170.7, 169.7, 169.1, 167.6, 167.4, 164.3, 159.9, 157.1, 156.4, 155.6, 155.1, 152.2, 151.3, 150.3, 150.1, 148.3, 146.4, 142.4, 139.8, 137.0, 136.6, 135.74, 135.66, 134.2, 133.8, 132.0, 131.2, 130.9, 128.7, 127.4, 127.2, 127.1, 126.3, 125.5, 124.7, 123.4, 121.7, 118.0, 116.5, 116.2, 107.3, 106.0, 104.6, 102.2, 101.2, 97.6,

78.0, 77.0, 76.7, 71.6, 71.4, 71.1, 70.3, 63.5, 62.2, 61.7, 61.3, 58.1, 57.0, 54.9, 53.7, 53.6, 51.0, 44.6, 41.0, 36.9, 35.4, 33.7, 24.2, 23.0, 22.5, 21.5, 20.6, 18.8, 17.4. **MS (MALDI):** m/z =1738.51, calcd for $C_{80}H_{87}N_{13}O_{26}Cl_2Na$ $(M+Na)^+$:1738.52. **HRMS (ESI):** m/z = 1738.51510, calcd for $C_{80}H_{87}N_{13}O_{26}Cl_2Na$ $(M+Na)^+$: 1738.51545. **HPLC (method B, UV detection, 282 nm):** 3.090 min.

IV. NMR, MS, and HPLC Data

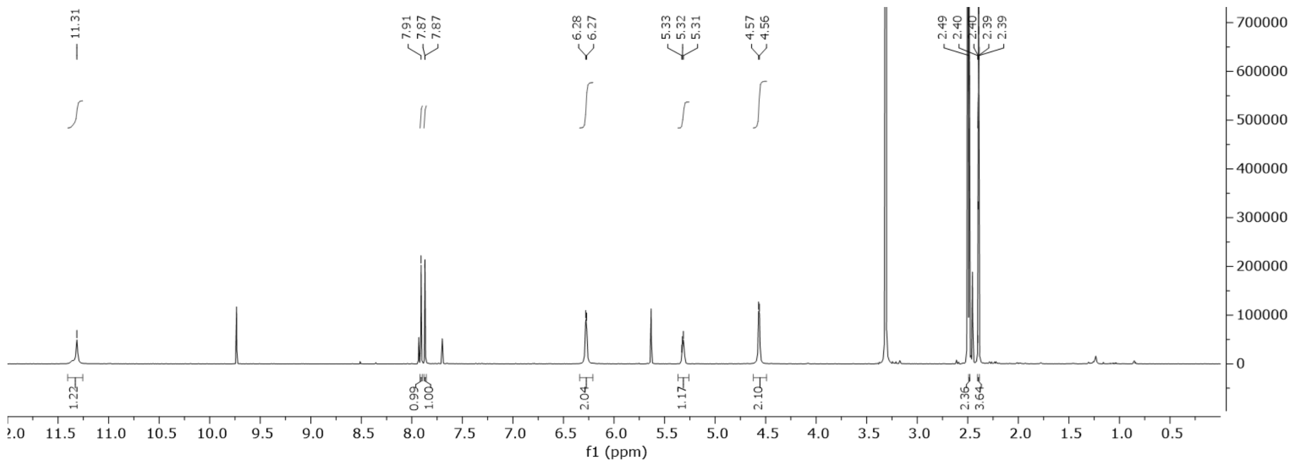

Figure S17. ^{1}H NMR spectrum of the intermediate gem-diol isolated during the synthesis of aldehyde **1**, recorded at 601 MHz in DMSO-d_6.

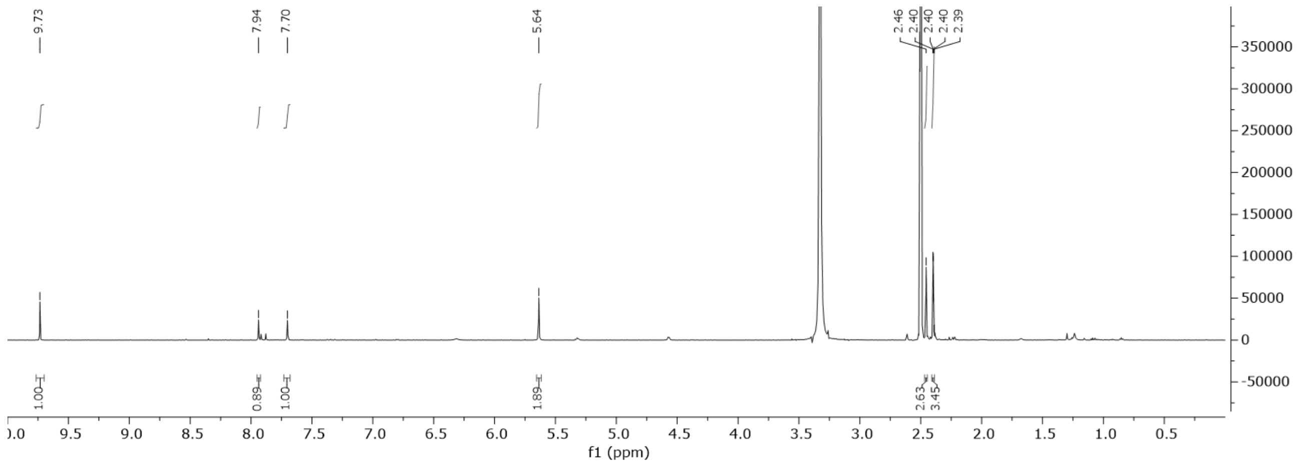

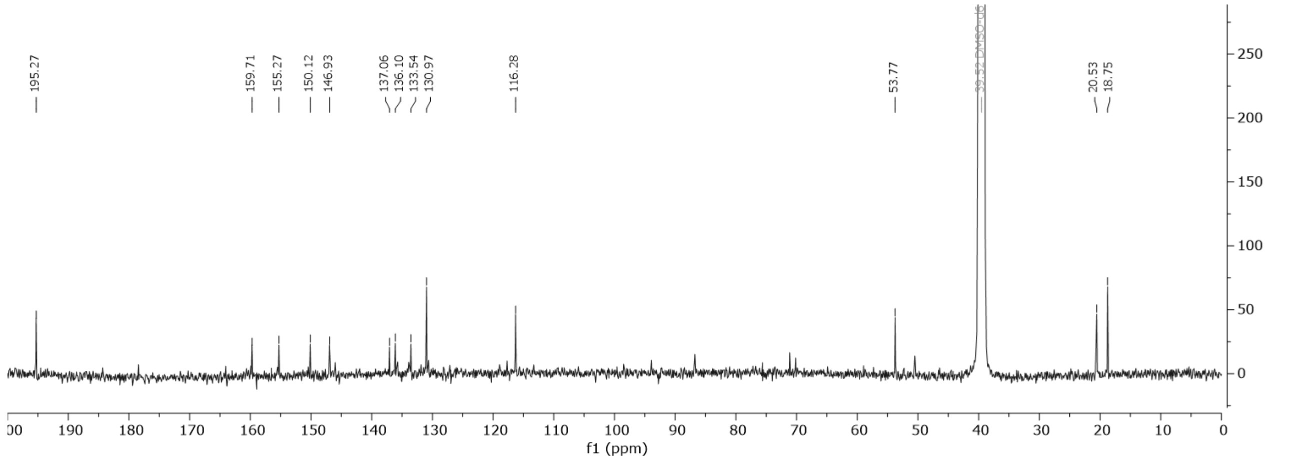

Figure S18. ^{1}H and ^{13}C NMR spectra of compound **1**, recorded at 601 MHz and 126 MHz respectively, in DMSO-d_6.

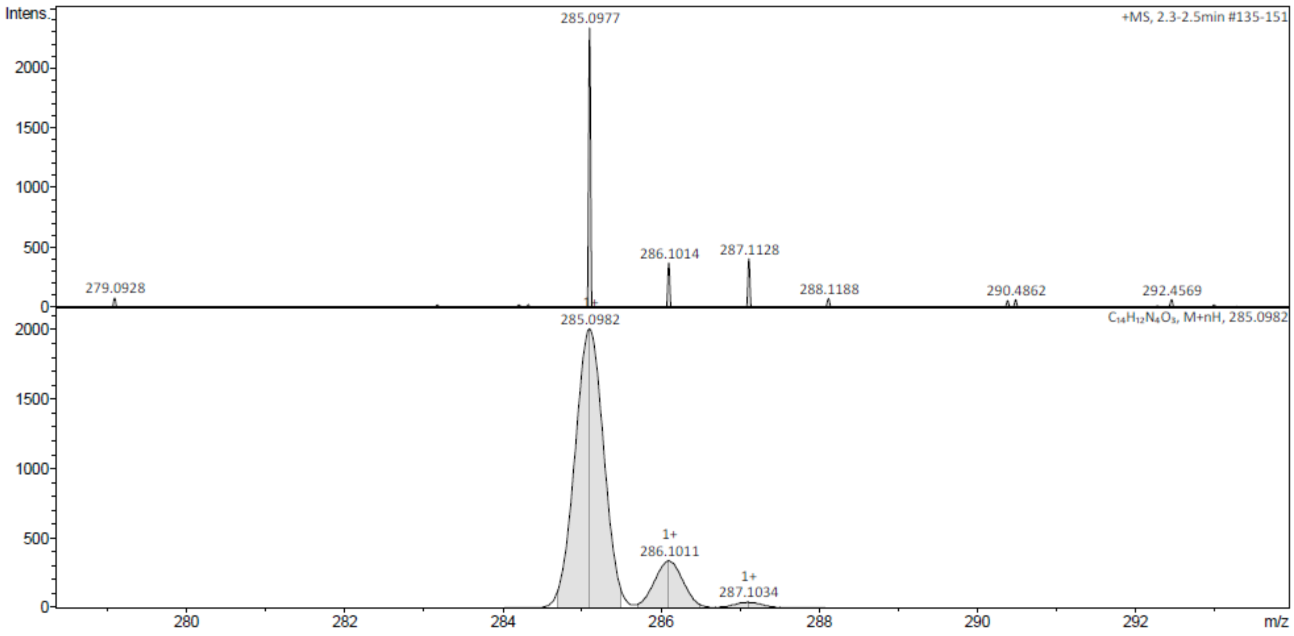

Figure S19. HRMS (ESI) spectrum for compound **1**.

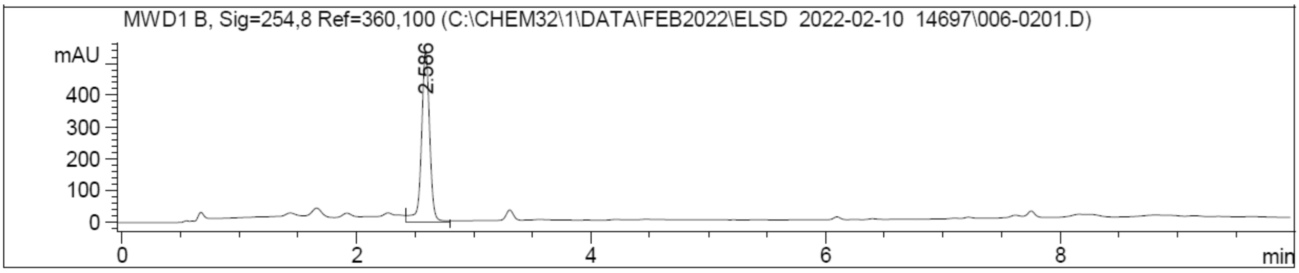

Figure S20. HPLC trace (detection at 254 nm) for compound **1**.

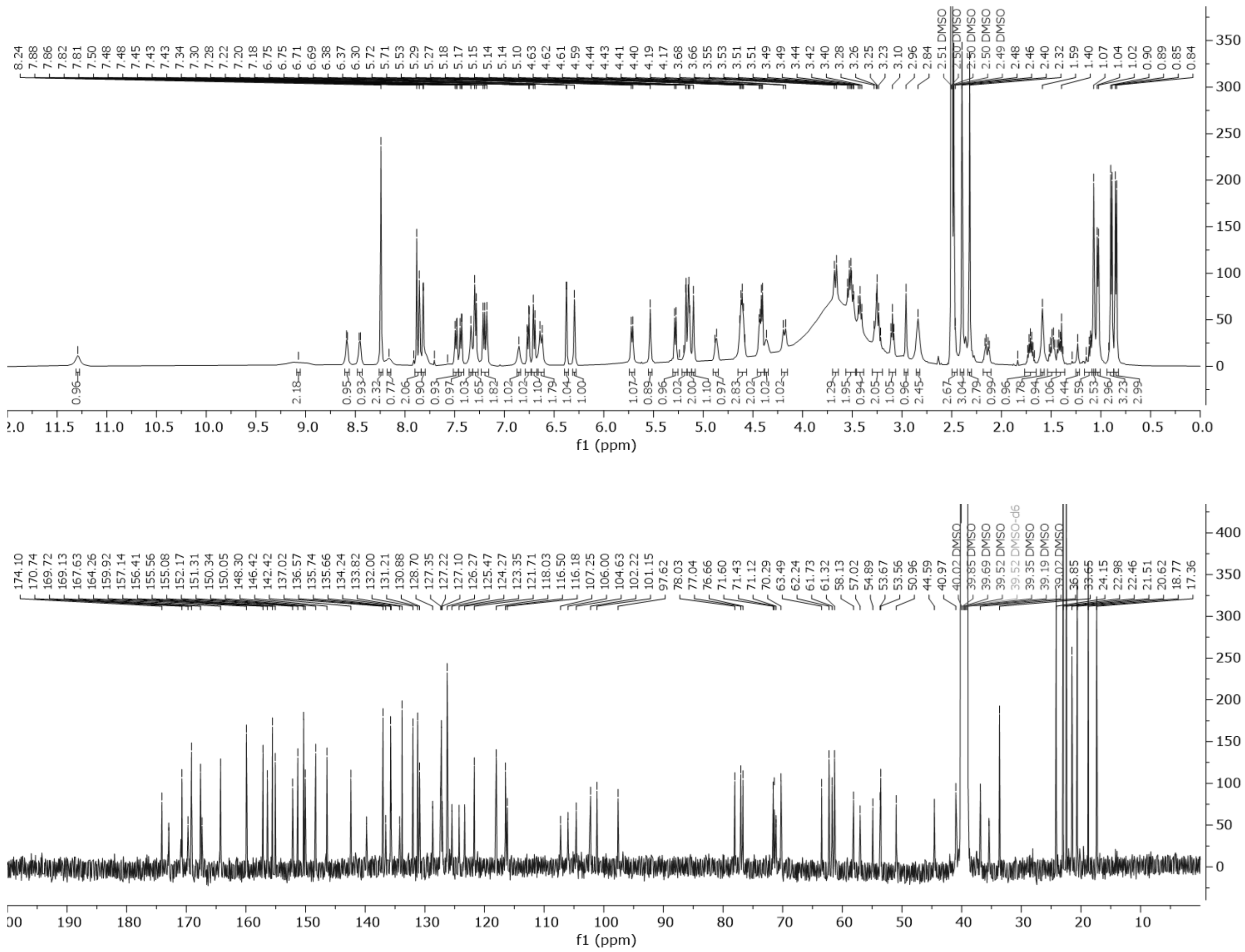

Figure S21. ^{1}H and ^{13}C NMR spectra of compound **VanB2**, recorded at 500 MHz and 126 MHz respectively, in DMSO-d_6.

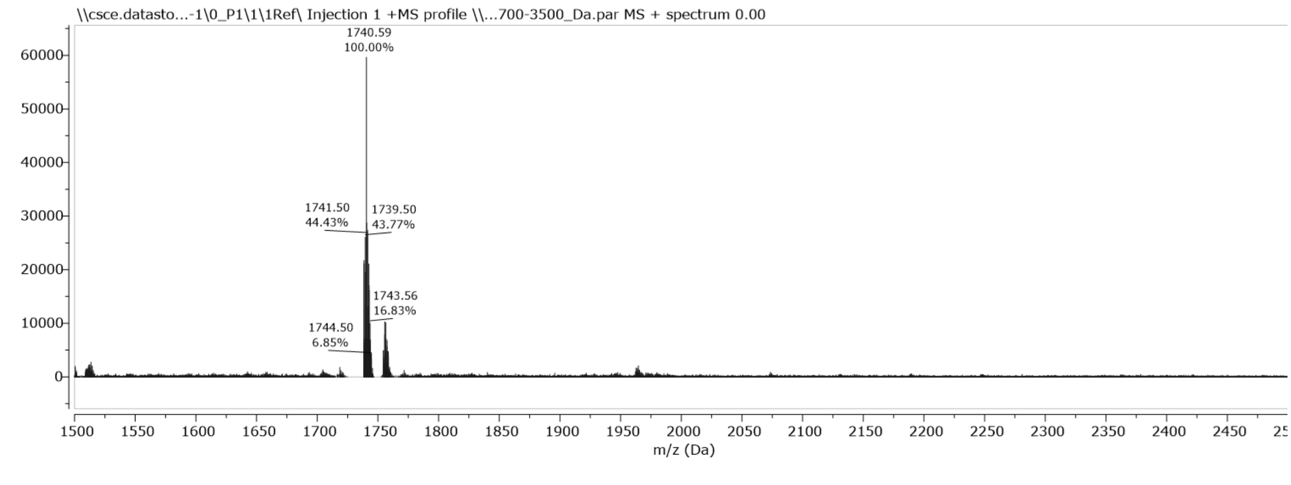

Figure S22. HRMS (MALDI-ToF) spectrum for **VanB2**.

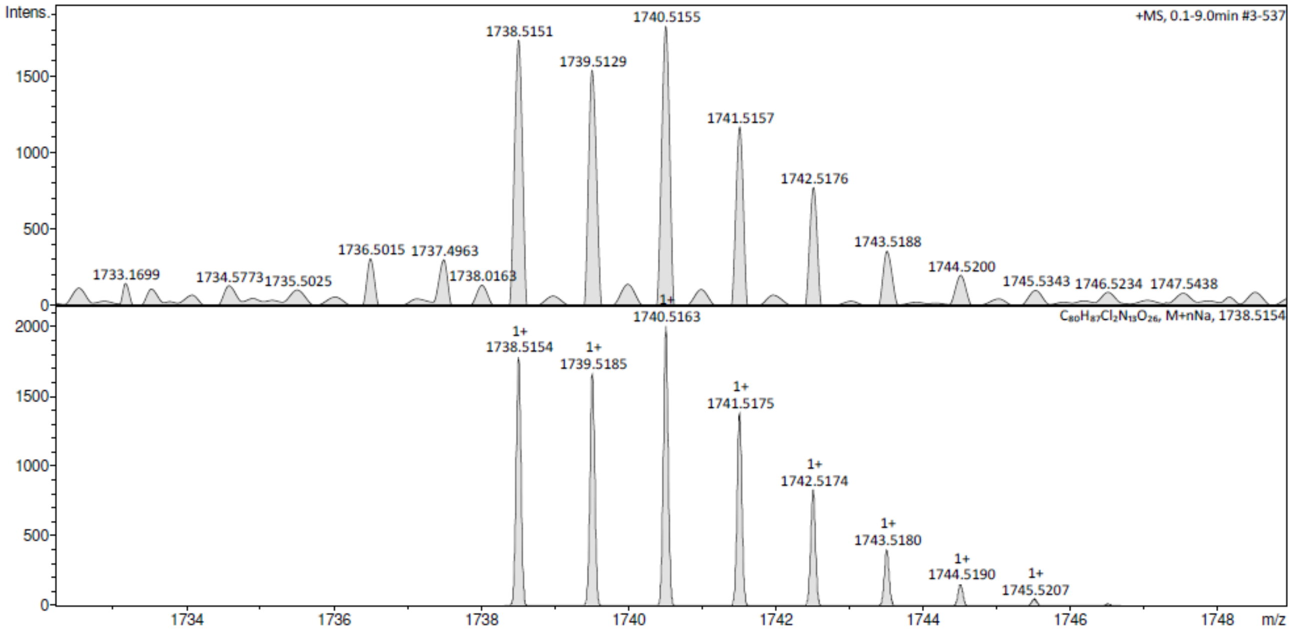

Figure S23. HRMS (ESI) spectrum for **VanB2**.

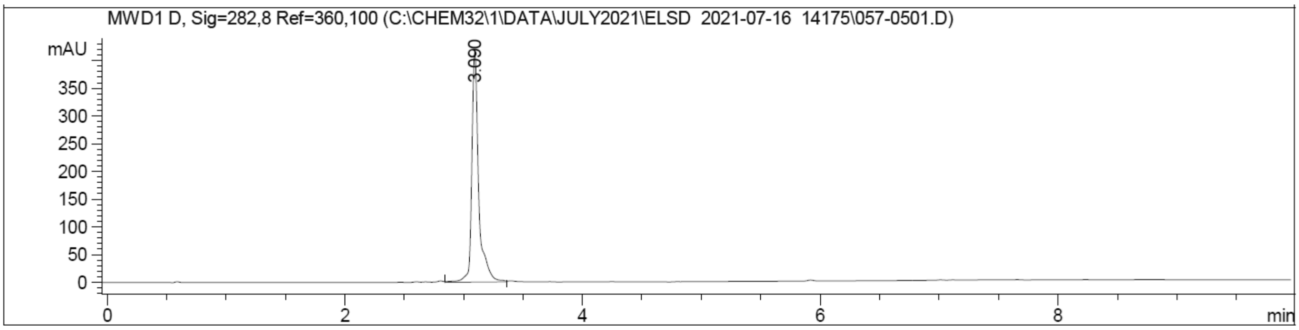

Figure S24. HPLC trace (detection at 282 nm) for **VanB2**.

V. References

1 C. Würth, M. Grabolle, J. Pauli, M. Spieles and U. Resch-Genger, *Nature Protocols*, 2013, **8**, 1535–1550.
2 U. Resch-Genger and K. Rurack, *Pure Appl. Chem., PAC*, 2013, **85**, 2005–2013.
3 A. M. Brouwer, *Pure Appl. Chem., PAC*, 2011, **83**, 2213–2228.
4 N. Adarsh, R. R. Avirah and D. Ramaiah, *Org. Lett.*, 2010, **12**, 5720–5723.
5 J. Baier, T. Maisch, M. Maier, E. Engel, M. Landthaler and W. Bäumler, *Biophysical Journal*, 2006, **91**, 1452–1459.
6 J. N. Chacon, J. McLearie and R. S. Sinclair, *Photochem Photobiol*, 1988, **47**, 647–656.
7 T. Furuta, S. S.-H. Wang, J. L. Dantzker, T. M. Dore, W. J. Bybee, E. M. Callaway, W. Denk and R. Y. Tsien, *PNAS*, 1999, **96**, 1193–1200.
8 C. G. Hatchard, C. A. Parker and E. J. Bowen, *Proceedings of the Royal Society of London. Series A. Mathematical and Physical Sciences*, 1997, **235**, 518–536.
9 M. Montalti, A. Credi, L. Prodi and M. T. Gandolfi, in *Handbook of Photochemistry*, CRC Press, 3rd edn., 2006.
10 S.-I. Murahashi, D. Zhang, H. Iida, T. Miyawaki, M. Uenaka, K. Murano and K. Meguro, *Chem. Commun.*, 2014, **50**, 10295–10298.
